# Reevaluating Assembly Evaluations with Feature Response Curves: GAGE and Assemblathons

**DOI:** 10.1371/journal.pone.0052210

**Published:** 2012-12-28

**Authors:** Francesco Vezzi, Giuseppe Narzisi, Bud Mishra

**Affiliations:** 1 School of Computer Science and Communication, KTH Royal Institute of Technology, Science for Life Laboratory, Solna, Sweden; 2 Cold Spring Harbor Laboratory, Cold Spring Harbor, New York, United States of America; 3 Courant Institute of Mathematical Sciences, New York University, New York, New York, United States of America; 4 NYU School of Medicine, New York University, New York, New York, United States of America; University of Chicago, United States of America

## Abstract

In just the last decade, a multitude of bio-technologies and software pipelines have emerged to revolutionize genomics. To further their central goal, they aim to accelerate and improve the quality of *de novo* whole-genome assembly starting from short DNA sequences/reads. However, the performance of each of these tools is contingent on the length and quality of the sequencing data, the structure and complexity of the genome sequence, and the resolution and quality of long-range information. Furthermore, in the absence of any metric that captures the most fundamental “*features*” of a high-quality assembly, there is no obvious recipe for users to select the most desirable assembler/assembly. This situation has prompted the scientific community to rely on crowd-sourcing through international competitions, such as Assemblathons or GAGE, with the intention of identifying the *best* assembler(s) and their features. Somewhat circuitously, the only available approach to gauge *de novo* assemblies and assemblers relies solely on the availability of a high-quality fully assembled reference genome sequence. Still worse, reference-guided evaluations are often both difficult to analyze, leading to conclusions that are difficult to interpret. In this paper, we circumvent many of these issues by relying upon a tool, dubbed 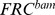, which is capable of evaluating *de novo* assemblies from the read-layouts even when no reference exists. We extend the FRCurve approach to cases where lay-out information may have been obscured, as is true in many deBruijn-graph-based algorithms. As a by-product, FRCurve now expands its applicability to a much wider class of assemblers – thus, identifying higher-quality members of this group, their inter-relations as well as sensitivity to carefully selected *features*, with or without the support of a reference sequence or layout for the reads. The paper concludes by reevaluating several recently conducted assembly competitions and the datasets that have resulted from them.

## Introduction

The extraordinary advances in Next Generation Sequencing (NGS) technologies over the last ten years have triggered an exponential drop in sequencing cost, thus making it possible to perform whole-genome shotgun (WGS) sequencing of almost every organism in the biosphere. In particular, recent WGS projects are distinctive by the way they have facilitated whole genome sequencing at a high coverage (*i.e.*, higher than 50×), albeit, composed of relatively short sequences (*i.e.*, reads).

Despite this impressive progress, recent efforts have underlined the difficulties in trading-off read length against read coverage. It is now well recognized how the short reads have made the assembly problem significantly harder [1] owing to the complexity involved in resolving (*i.e.*, span over) long repeats.

Nonetheless, this challenge has been confronted recently with sophisticated and novel techniques, embedded in a diverse set of tools all aiming to solve *de novo* assembly problem. Such tools (*i.e.*, *assemblers*) are based on the simple assumption that if two reads share a sufficiently long subsequence then they are likely to belong to the same location in the genome. In order to represent and efficiently use such information for myriads of short reads, assemblers typically rely on compressed graph structures (often de-Bruijn graphs but also string-graphs). Moreover, additional heuristics are employed for error correction and read-culling.

More than twenty different assemblers have been designed to tame the computational complexity of assembling NGS reads, with the vast majority of them specifically targeting Illumina reads. One of the main consequences of this proliferation in software production is the difficulty in selecting one assembler over another, which often makes a Buridan’s ass of a bioinformatics researcher: Their effort spent on selecting the best assembler (*i.e.*, the largest haystack for the ass) ultimately diverts them from their real objective of answering biological questions (*i.e.*, leading to a disoriented and starving ass).

Adding to the confusion, every new genome presents its own sets of problems, *e.g*, ploidy, heterozigosity, repetitive structures, *etc*. The available assemblers usually are able to efficiently solve only some of these problems or are specifically designed for limited datasets (*e.g.*, bacterial genomes). A widely followed approach is to use multiple assemblers, run with different parameters, producing statistics that could point to the best among them. However, no clear way to select the “best” assembler has yet made itself obvious. As noticed by Miller in [2] all new published assemblers have been compared to the then-existing tools showing, every time, their better performances on a specific dataset and on some specific metrics. More often than not, only traditional metrics (*i.e.*, contiguity-based metrics) are used in comparing assemblers’ performances (*e.g.*, number of contigs, NG50, *etc.*) – a strategy that suffers from the drawback of emphasizing only assembly size. Moreover, in [3], NG50 (the most “abused” metric) has been demonstrated to be a bad assembly quality predictor. In contrast, more reliable results can be produced, when a reference sequence is available, since contigs could be aligned against it in order to judge the number of errors (*i.e.*, reference-based metrics). Unfortunately, currently, no effort is usually made in weighting or scoring qualitatively different types of errors, thus reducing this approach to a simple error counting without accounting for subtle differences among the different types of errors.

More recently, the focus has shifted from seeking just contiguity to assembly precision. An earlier study [4] showed that in the published and revised human genome [5] on average 10% of assembled fragments were assigned the wrong orientation and 15% of fragments, placed in a wrong order. Recall that this draft sequence of the Human Genome [5], which was released in 2001, had taken several large teams more than five years to finish and validate (but only at a genotypic level). With many projects left at draft level, NGS technologies have worsened this situation even further. Alkan in [6] criticized two of the major NGS achievements: the assembly of the Han Chinese and Yoruban individuals [7] both sequenced with Illumina reads. Alkan identified 420 Mbp of missing repeat sequences from the Yoruban assembly, and estimated that in both assemblies almost 16% of the genome was missing.

Despite these widely discussed and obvious problems, there still persists a lack of standard procedures and methods to validate and evaluate assemblies. Several projects have been initiated to explore the parameter space of the assembly problem, in particular in the context of short read sequencing [6], [8], [9].

Recently, a growing number of studies have aimed at independently evaluating different assemblers or assembly pipelines. *Assemblathon 1* [10] and *Assemblathon 2* sought to assess assemblers’ performances on common datasets encouraging a competition among researchers/users and assemblers’ developers. In its earliest version, the competition was performed on a simulated dataset, leaving open to a criticism of the effectiveness of its genome and read simulators [3]. Assemblathon 1′s entries were evaluated and ranked, based on a mixture of contiguity-based and reference-based metrics. The final result is a large table (see Table 3 in [10]) in which some assemblers perform well on some metrics while behaving poorly on others, thus, leaving its interpretation somewhat equivocal.

A similar but independent study, dubbed GAGE, has been designed to critically evaluate and compare assemblers on four different large-scale NGS projects [11]. The presence of an already assembled reference sequence for three of the studied genomes allowed the authors to assess assembly quality. One of the main message of this study is that the same assembler can produce utterly different qualities of results on different datasets. Moreover, Salzberg and colleagues showed how assemblers’ performance is affected by data quality: preprocessing used in read correction seems fundamental to improve assemblers’ results. The main conclusion of this study is that there is no universal “assembly recipe” to be used for assembling new genomes. An assembler working well on certain genomes may exhibit drastically poorer performance when used to assemble even a fairly similar genome. A fundamental criticism against GAGE is that each assembler was tuned to maximize the resulting NG50 for each dataset. This was done to mimic the behavior of typical users, but builds on an extremal statistic which, as mentioned earlier, also happens to be the worst quality predictor [3]. Only after this tuning step assembly quality was measured by comparison to a reference. As in Assemblathon 1, GAGE output is presented as a set of tables with massive amount of–often hard-to-interpret–information.

This state of affairs is not completely surprising, given the complexity of assembly evaluation, especially, when all errors cannot be substantially eliminated. For instance, even after six months since Assemblathon 2′s competition, an official ranking remains undisseminated (except for the one based on NG50).

Recently, Narzisi and Mishra in [12] proposed a new metric, Feature Response Curve (FRCurve), capable of capturing the trade-off between contigs’ contiguity and correctness. FRCurve is based on the principle that the assembly precision can be predicted by identifying on each contig a set of suspicious regions (*i.e.*, *features*): contigs are then sorted from the longest to the shortest, and for each feature threshold 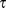 only the longest contigs whose total sum of features is less than 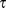 are used to compute the genome coverage (*i.e.*, a single point in the FRCurve). Such technique has been extensively studied and evaluated in [3]. Despite its power the main limitation of FRCurve is that it requires the so-called *read layout*, a standard output of Sanger-based assemblers, but missing in the vast majority of NGS assemblers. Such dependency restricts FRCurve analysis tools to only OLC, overlap-layout-consensus based assemblers and thus to a limited subset of NGS-based studies.

In this paper, we present an enhanced tool, named 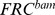, capable of computing FRCurve from the alignment of the reads to the assembled contigs. In particular, we show that this method is able to correctly and rigorously evaluate assemblers’ performance and precision, even in the absence of a reference sequence, while using a broad set of metrics, not just those based on assembly contiguity. We begin by describing the set of implemented features, and then evaluate our tool on the datasets used in the three major assembly evaluations efforts: GAGE, Assemblathon 1 and Assemblathon 2.

## Materials and Methods

Almost always, *de novo* assembly is carried out using more than one library. In the Illumina scenario we typically have at least two libraries: one paired-end library (PE), and one mated-pair library (MP). The former provides paired reads in the standard orientation (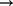 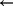) with insert size that can vary between 150 bp (overlapping fragments) and 1000 bp (standard PE). The latter yields pairs of sequences in the opposite direction (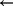 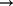) and the insert size is much longer (usually in the range between 3 and 10 Kbp). Due to the different cost of the two protocols a typical sequencing project consists of one high coverage PE library and one low coverage MP library. The main advantage of MP reads is to improve contiguity through scaffolding and gap-filling procedures. However, the MP library is intrinsically more difficult to obtain than standard PE libraries and are usually affected by redundancy (PCR duplicates) and uneven genome representation.

After PE reads and MP reads are aligned against the assembly itself, the ordered and indexed BAM files are input into 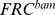. 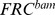 needs at least one PE library and, if available, one MP library. The user needs to provide a rough estimation of the insert size and of the standard deviation for both libraries and an estimation of the genome length. Read coverage and spanning coverage are computed directly from the BAM files.

Several features are computed in order to identify problems related to read coverage, mate pair happiness [8], and compression/expansion events (*i.e.*, CE-statistics) [13]. As a consequence of their different nature, PE reads and MP reads are used to compute two different sets of features. The former is used to compute the following features: LOW_COV_PE, HIGH_COV_PE, LOW_NORM_COV_PE, HIGH_NORM_COV_PE, COMPR_PE, STRECH_PE, HIGH_SINGLE_PE, HIGH_SPAN_PE, and HIGH_OUTIE_PE. The latter library is used to compute only a subset of the features, similar to the ones in the previous set: COMPR_MP, STRECH_MP, HIGH_SINGLE_MP, HIGH_SPAN_MP, and HIGH_OUTIE_MP. The main difference is due to the fact that MP reads usually provide a low read coverage (*i.e.*, vertical) but produce a high spanning coverage (*i.e.*, horizontal). Therefore MP reads are best used to compute features related to long range information (see Table 1 and Document S1 for a detailed description of features).

**Table 1:** Description of implemented features.

Feature	Description
LOW_COV_PE	*low read coverage* areas (all aligned reads).
HIGH_COV_PE	*high read coverage* areas (all aligned reads).
LOW_NORM_COV_PE	*low paired-read coverage* areas (only properly aligned pairs).
HIGH_NORM_COV_PE	*high paired-read coverage* areas (only properly aligned pairs).
COMPR_PE	*low CE-statistics* computed on PE-reads.
STRECH_PE	*high CE-statistics* computed on PE-reads.
HIGH_SINGLE_PE	*high number of PE reads with unmapped pair*.
HIGH_SPAN_PE	*high number of PE reads with pair mapped in a different contig/scaffold*.
HIGH_OUTIE_PE	*high number of mis-oriented or too distant PE reads*.
COMPR_MP	*low CE-statistics* computed on MP reads.
STRECH_MP	*high CE-statistics* computed on MP reads.
HIGH_SINGLE_MP	*high number of MP reads with unmapped pair*.
HIGH_SPAN_MP	*high number of MP reads with pair mapped in a different contig/scaffold*.
HIGH_OUTIE_MP	*high number of mis-oriented or too distant MP reads*.

The Table provides a brief description for each implemented feature.


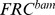
 outputs several files: (*a*) the FRCurve itself (to be plotted), (*b*) the FRCurves for each individual feature, and finally, (*c*) a position-by-position description of the feature (in GFF format). This last file holds for each contig the identified features, together with the start and end points.

### Datasets

For comparative analysis of NGS assemblers, both GAGE and Assemblathon studies offer state-of-the-art datasets, which could also be re-purposed to evaluate reliability of the new 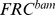. These datasets were of particular interest to us for several reasons, falling into three categories: (*i*) datasets consist of state-of-the-art sequences, with reads often belonging to several paired-end and mate-pairs libraries; (*ii*) availability of already “optimized” assemblies; (*iii*) presence of a reference sequence for most of the sequenced organism.

The first category allowed us to test 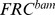 against state-of-the-art datasets and to take advantage of different insert-types. The second category enabled us to use assemblies that may be considered as the “best” achievable, since they were obtained by *de novo* assembly experts (*i.e.*, GAGE) or by the same assemblers’ developers (*i.e.*, Assemblathon). Specifically, the availability of a reference sequence, allow us to measure assemblies’ correctness, thus also demonstrating how 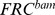 and the computed features are able to effectively gauge assembly accuracies and to identify suspicious regions (*i.e.*, mis-assemblies).

In total, we tested 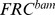 on five datasets: *Staphylococcus aureus*, *Rhodobacter sphaeroides*, and Human chromosome 14 from GAGE, data of simulated genomes from Assemblathon 1 competition, and *Boa constrictor* (*i.e.*, Snake) from Assemblathon 2 competition. All five datasets are composed of high coverage (*i.e.*, all exceeding 40×) Illumina paired-end and mate-pair read-libraries. *S. aureus* has been assembled with 7 different assemblers (see Table 2), *R. sphaeroides* and Human chromosome 14 (hereafter Hc14) have been assembled with 8 different assemblers (see Tables 3 and 4). Assemblathon 1 and Assemblathon 2 comprise 59 and 12 entries respectively. The large number of Assemblathon 1 entries is simply a consequence of the rule to permit multiple submissions: we decided to download only the best entry from each team, as determined by the Assemblathon 1 ranking (refer to [10] for more details), for a total of 17 entries. Summarizing, we tested 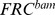 on five extremely different datasets for a total of 43 assemblies.

**Table 2:** *Staphylococcus aureus* (GAGE) assembly evaluation and features estimation.

assembler	Ctg (Kbp)	NG50	Chaff (%)	Indels	Misjoins	Inv	Reloc	Sens	Spec
ABySS	246	34	6.66	10	6	4	2	99.25	62.70
Allpaths-LG	**12**	1,092	0.03	12	**4**	**0**	4	84.79	89.97
Bambus2	17	1,084	0.00	215	14	2	12	97.14	83.51
MSR-CA	17	**2,412**	0.00	14	15	9	6	88.12	92.89
SGA	546	208	0.00	**4**	**4**	1	**3**	95.48	63.71
SOAPdenovo	99	3312	0.35	36	25	2	23	95.32	86.69
Velvet	45	762	0.41	16	31	10	21	96.83	84.26

For each assembler we report the number of contigs/scaffolds produced (Ctg), the NG50, the percentage of short (Chaff) contigs (the percentage is computed with respect to the real genome length), the number of long (*i.e.*, >5 bp) indels (Indels), the number of Misjoins, the number of inversions (Inv), the number of relocations (Rel), the features sensitivity (Sens), and the features specificity (Spec).

**Table 3:** *Rhodobacter sphaeroides* (GAGE) assembly evaluation and features estimation.

assembler	Ctg (Kbp)	NG50	Chaff (%)	Indels	Misjoins	Inv	Reloc	Sens	Spec
ABySS	1701	9	1.59	38	24	2	22	98.92	37.26
Allpaths-LG	**34**	**3,192**	0.01	37	6	**0**	6	90.73	93.36
Bambus2	92	2,439	0.00	378	5	0	7	75.84	82.76
CABOG	130	66	0.00	24	15	5	10	89.04	82.51
MSR-CA	43	2,976	0.00	31	15	3	12	87.87	93.92
SGA	2096	51	0.00	**4**	**4**	**0**	**4**	96.66	62.89
SOAPdenovo	166	660	0.44	431	11	1	10	92.90	86.62
Velvet	178	353	0.48	27	21	6	15	92.04	83.33

For each assembler we report the number of contigs/scaffolds produced (Ctg), the NG50, the percentage of short (Chaff) contigs (the percentage is computed with respect to the real genome length), the number of long (*i.e.*, >5 bp) indels (Indels), the number of Misjoins, the number of inversions (Inv), the number of relocations (Rel), the features sensitivity (Sens), and the features specificity (Spec).

**Table 4:** Human chromosome 14 (GAGE) assembly evaluation and features estimation.

assembler	Ctg (Kbp)	NG50	Chaff (%)	Indels	Misjoins	Inv	Reloc	Sens	Spec
ABySS	51301	2,1	34.78	762	**22**	**15**	**7**	95.83	18.79
Allpaths-LG	**225**	**81,647**	0.02	2575	146	44	102	68.46	96.79
Bambus2	1792	324	0.00	5651	3409	1759	1650	86.26	55.04
CABOG	479	393	0.00	2894	746	435	311	62.19	95.92
MSR-CA	1425	893	0.01	3097	2311	83	1439	86.10	84.71
SGA	30975	83	0.00	**681**	150	90	60	92.13	65.38
SOAPdenovo	13501	455	3.09	3902	1529	537	992	90.59	73.10
Velvet	3565	1,190	4.23	4172	9525	4023	5502	91.60	67.55

For each assembler we report the number of contigs/scaffolds produced (Ctg), the NG50, the percentage of short (Chaff) contigs (the percentage is computed with respect to the real genome length), the number of long (*i.e.*, >5 bp) indels (Indels), the number of Misjoins, the number of inversions (Inv), the number of relocations (Rel), the features sensitivity (Sens), and the features specificity (Spec).

For each dataset we selected one paired-end library and one mate-pair library (see Document S1 for more details). These two libraries were then aligned against the available assemblies using rNA [3]. We aligned reads using also BWA [14] without detecting any noticeable difference (see Document S1).

Using libraries with different insert sizes (*i.e.*, paired-end and mate-pair reads) enabled us to identify different features types. On the one hand, paired-end reads, characterized by a short insert size (*i.e.*, usually less than 600 bp) are able to highlight local mis-assemblies and relatively small insertions/deletions events. On the other hand, mate-pairs, characterized by a larger insert size (*i.e.*, usually more than 2 Kbp) are able to highlight larger insertion/deletion events and larger mis-assemblies (*e.g.*, scaffolding errors).

## Results

**Figure 1:**
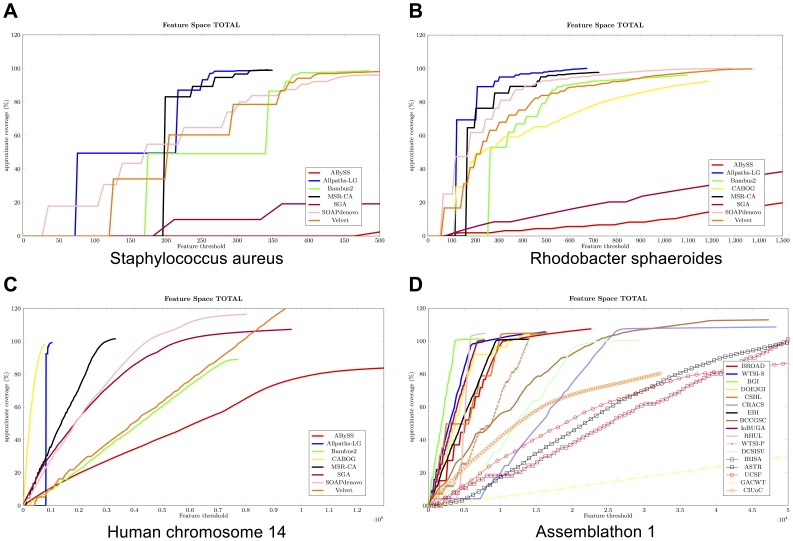
FRCurve computed on the three GAGE datasets and on Assemblathon 1 entries. Figures A, B, and C show the FRCurves computed on the three GAGE datasets (*Staphylococcus aureus*, *Rhodobacter sphaeroides*, and Human chromosome 14). Figure D shows the FRCurves computed on Assemblathon 1 entries.

Figure 1 shows FRCurves for the three GAGE genomes (*S. aureus* Figure 1A, *R* *. spheroides* Figure 1B, and Hc14 Figure 1C) and for Assemblathon 1 entries (Figure 1D). For each of the analyzed assemblies we aligned contigs against the reference genome. To accomplish this task we employed the scripts available on GAGE website [11]. Assembly statistics are reported in Tables 2, 3, 4, and 5.

**Table 5:** Assemblathon 1 assembly evaluation and features estimation.

assembler	Ctg (Kbp)	NG50	Chaff (%)	Indels	Misjoins	Sens	Spec
BROAD	989	**8,396**	0.00	903	236	92.99	93.88
BGI	1897	1,716	0.26	994	656	81.39	97.48
WTSI-S	1380	2,874	0.00	**132**	197	95.10	96.55
DOEJGI	771	9,073	0.03	163	**181**	94.32	96.80
CSHL	1842	3,254	3.05	3704	733	90.76	95.18
CRACS	6165	2,712	0.00	319	990	96.59	83.27
BCCGSC	3314	825	2.92	488	636	96.69	88.97
EBI	2173	959	0.39	674	1021	78.66	94.24
IoBUGA	**467**	1,801	0.18	3596	1249	71.65	94.16
RHUL	4999	43	0.00	336	1040	91.70	95.88
WTSI-P	1448	502	0.00	4121	2389	93.53	89.94
DCSISU	4790	315	0.00	1284	2366	90.14	79.60
IRISA	3539	1,406	0.05	2518	350	95.28	76.90
ASTR	6228	57	0.00	336	2265	91.79	69.97
UCSF	14821	22	0.00	12131	5127	93.85	66.19
GACWT	24297	9	0.00	2197	1487	94.10	49.36
CIUoC	14993	6	0.00	3215	1889	77.09	67.29

For each assembler we report the number of contigs/scaffolds produced (Ctg), the NG50, the percentage of short (Chaff) contigs (the percentage is computed with respect to the real genome length), the number of long (*i.e.*, >5 bp) indels (Indels), the number of Misjoins, the features sensitivity (Sens), and the features specificity (Spec).

The four tables (Tables 2, 3, 4, and 5) report for each assembly/assembler the number of contigs/scaffolds produced (Ctg), the NG50, the percentage of short (*i.e.*, less than 200 bp) contigs (the percentage is computed with respect to the real genome length), the number of long (*i.e.*, >5 bp) indels (Indels), and the number of Misjoins (as reported by GAGE and Assemblaton 1). Moreover, with access to *dnadiff* [8] we could identify regions of real mis-assemblies, thus enabling us to compute sensitivity and specificity of our features. Note that sensitivity is defined as the ratio between true positives (*i.e.*, positions marked as mis-assembled by *dnadiff* and labelled by one or more features), and its sum with false negatives (*i.e.*, positions marked as mis-assembled by *dnadiff* but not labelled by any feature). Specificity, in contrast, is the ratio between true negatives (*i.e.*, positions not marked as mis-assembled by *dnadiff* and not labelled by any feature) and its sum with false positives (*i.e.*, positions not marked as mis-assembled by *dnadiff* but labelled by one or more features). The first measure enables FRCurve to identify problematic areas, while the latter measure distinguishes non -problematic from problematic regions (*e.g.*, if a feature marks all position in an assembly the sensitivity will be 1, however the specificity is likely to be close to 0).

### GAGE

Figure 1A and Table 2 show the FRCurve and the reference guided validation of *S. aureus* GAGE’s dataset respectively. From Figure 1A MSR-CA and Allpaths-LG appear to be the best performing assemblers on such datasets (*i.e.*, the steepest curves). These two assemblers are closely followed by SOAPdenovo, Velvet, and Bambus2, while SGA and ABySS clearly show bad performance. Both sensitivity and specificity of reported features are high (last two columns of Table 2), thus demonstrating that 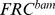 (and therefore our features) is able to correctly identify suspicious regions. Specificity is not particularly high only for ABySS and SGA. However, in these two assemblies the percentage of mis-assembled sequences identified by *dnadiff* are 20% and 8%, respectively, suggesting a high number of problematic regions close to the real mis-assembly sequences.

**Figure 2:**
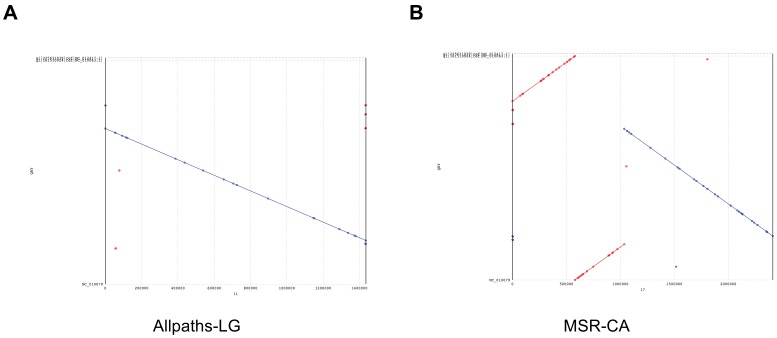
Dotplot validation of the longest scaffolds produced by Allpaths-LG and MSR-CA on *Staphylococcus a.* dataset. Figures A and B show the dotplot of the longest scaffolds produced by Allpaths-LG and MSR-CA against the reference genome.

Some remarks are warranted on the stepwise shape of some curves (*e.g.*, MSR-CA, Allpaths-LG and Bambus2). Such a shape indicates the presence of contigs with a large number of features that interrupts a smooth growth of the curve, which is particularly discernible when the number of contigs is low. As an example, consider the longest MSR-CA contig containing almost half of the features identified in the entire assembly. The high sensitivity and specificity reported in Table 2 show that these features represent truly problematic regions. Let us focus on Allpaths-LG and MSR-CA: in Figure 2 we present the alignment of the longest scaffold produced by Allpaths-LG and MSR-CA against the reference genome. From Figure 2B it is clear that the stepwise shape of MSR-CA’s FRCurve is a consequence of wrong choices made by the assembler. The situation is different in the Allpaths-LG case: Figure 2A shows a correctly reconstructed scaffold, therefore there is apparently no reason to justify the stepwise curve of Allpaths-LG. Puzzled by this anomaly, we plotted the FRCurve for each single feature (see Document S1). With this analysis, we discovered that Allpaths-LG has the best curve in the majority of the cases. However, there are two exceptions: STRECH_MP and COMPR_MP features, which are representative of compression or expansion events. Areas characterized by these features coincide with the circles in the dotplot (see Figure 2A): these areas involve small mis-joins (*i.e.*, less than 50 bases) or scaffold junctions (*i.e.*, sequences of Ns). A likely explanation is that such small mis-joins are able to “attract” reads that are responsible for the features. Moreover, STRECH_MP and COMPR MP features depend on CE statistics [13] and therefore on the choice of two thresholds, often estimated sub-optimally – note that, despite the availability of a reference sequence, these thresholds were estimated without it. MSR-CA is the assembly characterized by the largest number of areas composed of large numbers of mis-oriented mate/paired reads (*i.e.*, HIGH_OUTIE_PE and HIGH_OUTIE_MP), as a consequence of the large inversions and translocations present in the first scaffold. Other hints about MSR-CA’s problems come from the FRCurve obtained from the contigs (see Document S1): MSR-CA’s FRCurve is not as good as those of Allpaths-LG, SOAPdenovo and Bambus2.

This situation demonstrates that assembly evaluation is extremely difficult. With the help of a reference sequence it is clear that MSR-CA suffers from a large number of errors (see Table 2). However, in its absence, many users might have chosen MSR-CA over others, since it seemed to be able to reconstruct almost the whole genome with a single scaffold. FRCurve, without the use of a reference, was able to raise doubts about MSR-CA (*i.e.*, the only assembler with a high number of HIGH_OUTIE_PE and HIGH_OUTIE_MP features), thus suggesting a more careful manual validation on Allpaths-LG.

According to the FRCurve analysis, SGA (together with ABySS) is one of the worst performing assemblers. Although GAGE analysis concludes that SGA introduces relatively fewer errors, it is also the most fragmented one, consisting of 456 scaffolds (and 1252 contigs). This kind of assemblies, despite its low error-rate, tends to accumulate features related to copy number variation problems (*e.g.*, LOW_COV_PE) and features like HIGH_SPAN_MP suggesting problems in the scaffolding (*i.e.*, either errors in the scaffolding or a failure in establishing contig connections).

Similar analyses can be carried out for *R. sphaeroides* and Hc14 datasets whose FRCurves are represented in Figures 1B and 1C.

In *R. sphaeroides* dataset Allpaths-LG and MSR-CA again appear to be the two best performing assemblers, though SOAPdenovo, Velvet, and Bambus2 are not too far behind. The longest Allpaths-LG scaffold practically reconstructs the longest Rhodobacter chromosome: such scaffold contains only 100 features most of them suggesting the presence of regions affected by low paired read coverage (*i.e.*, LOW_NORM_COV_PE and LOW_COV_PE). Such features affect all others assemblers similarly. From FRCurve analysis one may conclude that Allpaths-LG is the best performing tool. The alignments of Allpaths-LG assembly against the reference further confirm this conclusion (see Document S1).

Bambus2 is characterized by a long (correct) scaffold that contains almost one third of its features. This situation is a consequence of regions composed of a large number of singleton reads (*e.g.*, HIGH_SINGLE_MP) and of areas suggesting the presence of compression events (*e.g.*, COMPR_MP). Similarly to the analysis of the *S. aureus*, these features seem to coincide with small gaps (as seen from the alignment of the longest Bambus2 scaffold against the reference sequence, see Document S1).

**Figure 3:**
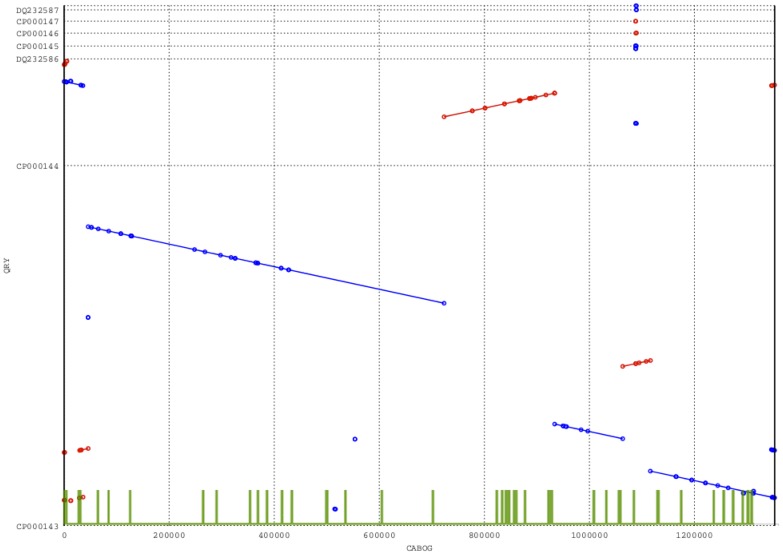
Dotplot validation of the longest scaffolds produced by CABOG on *Rhodobacter s.* dataset. Dotplot validation of the longest scaffold produced by CABOG on Rhodobacter dataset. The green lines represent the Features identified by 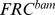.

From Figure 1B CABOG appears not to be a very well performing assembler. Such situation is confirmed by Figure 3 that shows the dotplot for CABOG’s longest scaffolds. The green columns at the bottom of the dotplot indicate the position where one or more features have been found by 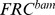. This plot shows how features are able to highlight problematic regions in the assembly, as the majority of them coincide with the mis-assemblies.

In the Hc14 case (see Figure 1C) Allpaths-LG and CABOG are clearly the best two assemblers. Allpaths-LG is the only assembler able to assemble almost all the sequences in a single scaffold containing, practically, all the features. The total number of features identified on this long scaffold is lower than the total amount of features identified in the 400 longest CABOG scaffolds. When we consider the FRCurves for each individual feature (see Document S1), we notice that Allpaths-LG longest contig is characterized by a large number of features suggesting coverage problems (*e.g*, LOW_NORM_COV_PE, LOW_COV_PE, HIGH_NORM_COV_PE, and HIGH_COV_PE features) and mated/paired read orientation problems (*e.g.*, HIGH_OUTIE_PE, and HIGH_OUTIE_MP features). As far as the coverage features are concerned, Allpaths-LG has almost always a lower number of such features than the other assemblers. Moreover, LOW_NORM_COV_PE feature is often the consequence of Allpaths-LG’s ability to correctly resolve repeated regions (pairs are not correctly aligned as a consequence of a repeat, see Document S1). Less straightforward is the explanation for the large number of features suggesting the presence of a large number of mis-oriented pairs (in this case Allpaths-LG being one of the worst assemblers). Such features are indicative of inversions and insertions events, although the dotplot shows an almost contiguous scaffold that reconstructs the Chromosome 14 without any particular problem (see Document S1). After a closer inspection, we discovered that such long scaffold is affected by a large number of small mis-joins as suggested by the circles in the main dotplot diagonal (see Document S1). We tested 10 different areas subject to such mis-joins and in all cases we discovered either a scaffold joint is too large or a scaffold joint has a short chimeric sequence in the middle, thus explaining the presence of a feature. The presence of these small mis-joins has been reported also in GAGE analysis: in the Hc14 dataset, the NG50 was close to 81 Mbp while the corrected-NG50 was 20 times shorter (the corrected-NG50 is the NG50 computed after breaking contigs at mis-assembled positions identified by the reference sequence). The low number of compression/expansion features (*i.e.*, CE statistics), as well as the low number of high-spanning and high-single reads related features in Allpaths-LG assembly (see FRCurves plots in Document S1) suggests that Allpaths-LG is able to return an assembly that is highly and correctly connected. However, the relatively large number of paired-end related features suggests the presence of small local mis-assemblies. On the other hand, CABOG produced a more fragmented assembly characterized by a small number of features. CABOG’s most frequent features (*i.e.*, HIGH_SPAN_PE and HIGH_SPAN_MP) suggest a systematic failure during the scaffolding phase in correctly merging contigs and inferring their order.

From FRCurve analysis alone, it is much harder to decide between the top two assemblers: Allpaths-LG and CABOG, though when the reference sequence is available, it is evident that Allpaths-LG suffers less from errors than CABOG (see Table 4). When considering only contigs (see Document S1) CABOG and Allpaths-LG still outperform other assemblers, as clearly proved by GAGE analysis (longest NG50).

With almost 30,000 features MSR-CA is the third ranking assembler as determined by the FRCurve analysis. MSR-CA is closely followed by SOAPdenovo and SGA. It is again difficult to fully ascertain such ranking, and even the reference guided validation in Table 4 does not lead to a clear and conclusive opinion. The majority of SGA’s features are a consequence of the highly fragmented assembly (see HIGH_SINGLE_MP FRCurve in Document S1). However the small number of errors (see Table 4) demonstrates that the final sequences are correct. SOAPdenovo is slightly better than MSR-CA as far as the number of errors is concerned, notwithstanding the fact that SOAPdenovo is more fragmented than MSR-CA. SOAPdenovo is particularly affected by the presence of mis-oriented paired reads (*i.e.*, HIGH_OUTIE_PE feature).

In all the three GAGE datasets the sensitivity of the 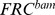 is almost always higher than 90% (CABOG is an exception, but it must be noted that the percentage of mis-assembled sequences is less than 1.4% of the genome length). Specificity is in general high, with the exception of assemblies characterized by high errors rates (*e.g.*, more than 40% of Velvet assembly is marked as suspicious by *dnadiff* on Hc14).

### Assemblathon 1

Assemblathon 1 dataset differs from that of GAGE mainly in two ways: it is much larger and it is obtained solely by simulation. Figure 1D and Table 5 summarize the analysis performed on such datasets. It is of particular interest to compare FRCurve assembly evaluation with Assemblathon 1 paper evaluation [10]. The order of the entries in Table 5 and of the legend in Figures 1D follows the Assemblathon 1 ranking.

Despite the presence of some outliers, the FRCurve analysis is close to the ranking obtained by Earl *et al.* BGI, WTSI-S, DOEGI, and CSHL were found by the FRCurve analysis to be better performing assemblers. They, together with Broad Institute’s (*i.e.*, Allpaths-LG), were the five best assemblers according to Assemblathon 1 ranking. A similar analysis could determine the worst performing assemblers. CIUoC, GACWT, UCSF, ASTR, and IRISA are clearly characterized by undesirable FRCurves (CIUoC’s long contigs contain few errors, even though the assembly contains only a fraction of the whole genome and small contigs contain many features).

There are some clear differences, for example Broad’s Allpaths-LG, the best assembler in Assemblathon 1 ranking is clearly among the best ones also in our FRCurve-based analysis, but has a high number of features suggesting problems with paired reads (*i.e.*, LOW_NORM_COV_PE and HIGH_SPAN_PE features). We discovered that these two features are highly correlated: in all the analyzed cases we discovered the presence of a small contig perfectly (or almost perfectly) aligning against a larger contig, probably the result of a wrong copy number estimation or of an unresolved allele splitting event. This observation is consistent with the analysis by Eearl *et al.*, as, for instance, Broad’s entry ranks 11*th* for copy number statistics.

Another clear difference is CRACS, the 6*th* ranking assembler in Assemblathon 1 evaluation, but an average performing assembler according to FRCurve analysis. The poor performance of this assembler is observed in a series of long contigs all exhibiting an extremely high coverage (*i.e.*, HIGH_COV_PE). This is clearly reflected also in the ranking given by Assemblathon 1: CRACS has clear problems in inferring copy number variation (12*th* ranking tool) and it reconstructs only 96% of the genome (14*th* ranking tool). FRCurve analysis suggests two possible solutions: either discard contigs strongly affected by this feature, or have CRACS developers reimplement an improved copy number variation estimation.

The last two assemblers we considered are RHUL and IoBUGA. Also in this case, these assemblers have FRCurves comparable to the best assemblers, but have been ranked below the median in Assemblathon-1′s evaluation. According to Assemblathon-1′s evaluation, RHUL has an acceptable number of substitutions (5*th* ranking tool); it is able to assemble sequences in the right copy number (5*th* ranking tool); and it is able to reconstruct (cover) the large part of the reference (4*th* ranking tool). However, it lacks good connectivity (13*th* ranking tool). FRCurve shows this assembler to contain most of its features in the longest scaffolds, while the short ones contain a small number of features. Note that the longest of RHUL ’s scaffolds generates a curve similar to ASTR’s. IoBUGA offers a similar story. Assemblathon-1′s ranking is difficult to interpret (15*th* ranking tool for substitutions and gene coverage but 3 *rd* ranking tool for copy number variation). This situation reemphasizes that reference guided validations are extremely difficult to interpret, especially when a tool exhibits contradicting performance. It should also be pointed out that IoBuga has the lowest sensitivity (see Table 5). It is clear that new features may be added in order to improve the effectiveness of 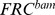 and FRCurve analysis. In this case, the availability of RNA-seq data may allow design of new features, capable of capturing assemblers’ ability to reconstruct gene expressions, splicing variants and intron-exon boundaries.

### Assemblathon 2

As shown earlier, the GAGE datasets were sufficient for testing the performance of 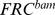 using only relatively small datasets. But with access to reference sequences, some of the limitations of the analysis became evident: only *S. aureus* and *R. spaeroides* are realistic datasets, while Hc14 has been partially simulated (reads have been aligned and extracted, see [11] for more details). Moreover, *S. aureus* and *R. spaeroides* datasets are extremely small in size and, to some extent, represent fairly easy-to-assemble genomes (*i.e.*, no heterozygosity or high ploidy). With access to Assemblathon 1 data, we further tested the 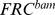 against a larger dataset that was previously analyzed and ranked. The main limitation of this dataset stemmed from the use of simulated reads, which often diverged from any reasonable model of reality.

**Figure 4:**
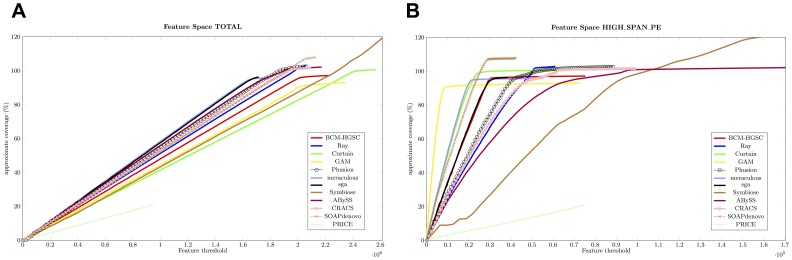
FRCurve computed on Assemblathon 2 entries. Figure A shows FRCurves for all the features, while Figure B shows the FRCurves plotted on a single feature (*i.e.*, High Spanning Paired Ends).

In order to show the applicability of our method to larger sequencing projects we tested the 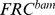 on all Assemblathon 2 entries for the Snake dataset (*Boa constrictor*). Results are shown in Figure 4. Surprisingly, when all features are considered all together, their FRCurves coincide closely with each other (see Figure 4A) suggesting that Assemblathon 2 participants, or the tools used by them, are converging to common results. We can identify two teams (assemblers) that are doing better than the others: SGA and Meraculous. There is a dense conglomerate of similarly behaving assemblers consisting of ABySS, Phusion, SOAPdenovo, CRACS, and Ray. Other assemblers appear less promising, though, except for the sole example of PRICE, none of them show unacceptably bad performance. The good performance of CRACS on this dataset brings to mind how drastically differently the same assembler could behave on different datasets.

Results are different if we concentrate on one feature at a time (see Figure 4B and Document S1). As an example, by inspecting the plot for the HIGH_SPAN_PE feature, we observe that GAM outperforms all the other assemblers. Meraculous and SGA show good performance too, together with Curtain, Symbiose, and BCM-HGSC. HIGH_SPAN_PE feature indicates presence of mis-joins, as often presumably close-by pairs are found in different contigs/scaffolds.

Particularly interesting is the FRCurve plot describing the presence of areas composed mainly of single ended reads (*i.e*, HIGH_SINGLE_MP feature, see Document S1). All assemblers are strongly affected by this feature demonstrating a general failure of all tools. A likely explanation is in a systematic failure in correctly assembling heterozygous *loci*, which generates holes in the assemblies, thus confounding the assemblers attempting to place both reads of mate-pairs. Note that this behavior is not present in the HIGH_SINGLE_PE features. A feature like HIGH_SINGLE_MP is clearly not informative in this dataset and may be ignored without affecting the analysis.

## Discussion

### Limitations of de novo Assembly Evaluation

The rapidly growing set of new assemblers aims to address the need for assembly tools capable of handling the vast amount of data produced by NGS (*e.g.*, Illumina) sequencers. This growth in data and tools, however, has led to another unmet need: a rigorous comparative study of these assemblers, which so far has only been carried out in a rather naïve way. Developers have focused more on performance (*e.g.*, RAM and CPU time) and connectivity (*e.g.*, contig number and NG50) rather than on correctness.

A commonly employed approach, currently being used to validate and gauge assemblies, is based on a *plethora* of *standard validation metrics*. We can identify four main groups: length-based statistics, reference-based statistics, simulation-based statistics, and long-range-information (LRI) based statistics.

Length-based statistics take into account only the size of the assembler output. These statistics comprise mean contig length, maximum contig length, and NG50. NG50, in principle, gives an idea of assemblies’ connectivity level. *All* length-based statistics are not linked to assembly correctness and emphasize only length: an assembler that eagerly merges together contigs can produce assemblies characterized by a large NG50 and by few long contigs. However, these long contigs are of no use if they contain too many misassemblies. NG50 has been shown in [3] to be a bad quality predictor. Nevertheless, length-based statistics are the basic, and some times the only, method used to judge assemblers performances, especially when the assembly tools are new [15], [16].

Assembly analysis would trivialize if the genome to be assembled was already available, which would make it possible to compare assemblers using only the reference-based statistics. The strategy would be to resequence an organism with an already available fully finished whole genome reference sequence. This approach would enable comparing assemblers from the computed real number of errors. The underlying premise is that good performances of an assembler on one dataset should reflect behavior on a wider range of datasets. However, studies like GAGE has shown that the same assembler can produce utterly different results on different genomes and different datasets – thus dashing any hope of generalizing the performance of a tool on the basis of a single dataset. Moreover, reference-metrics are in general difficult to interpret or, at least, are open to several interpretations: as an example reference-based metrics have been used both to demonstrate the high quality assembly of two human individuals in [7] as well as to demonstrate the opposite (their poor quality) in [6].

Simulation-based statistics face even more extreme hurdles: reads are simulated from a reference sequence and subsequently assembled. Vezzi *et al.* showed in [3] that simulated reads are likely to produce unrealistic contigs that cannot be used to judge assemblers’ performance. Despite these shortcomings, competitions like Assemblathon 1 have continued to use a simulation-based approach.

A more reasonable way to assess assembly correctness consists in the use of long range information. Second Generation Technologies are able to produce *mate-pairs*, that are pairs of reads at a mean distance of 2–8 Kbp. Mate-pairs play a crucial role in contig scaffolding, but they can be also used to gauge the assembly correctness: pairs should map on the assembly at the estimated distance and with the right orientation (depending on the sequencing technology being used). If such data is not used at assembly time it can be used as an external proof of correctness. A similar approach has already been applied with success in [8] (*i.e.*, mate-pair happiness). Other two commonly used LRI-methods are *physical maps* [17] and *optical maps* [18], [19]. Both rely on the relative locations of different genes and other DNA sequences of interest in the genome. Third Generation Sequencing Technologies (also known as Single Molecule Sequencing Technologies) and dilution-based sub-genomic sampling can also be used in the near future to estimate assembly correctness. The main drawback of LRI statistics is the fact that they require the production of new and often expensive data. Moreover, apart from the simple counting, it remains unclear how such information should be used to rank different assemblies that currently exist.

### FRCurve

The aim of this work is to present a new simple tool able to accurately evaluate assemblies and assemblers’ performance even in the absence of a reference sequence. Features have been first introduced in [8] to identify possible mis-assemblies. Narzisi and Mishra [9] used such features to compute the so called Feature Response Curve (FRCurve). FRCurve is closely connected to the standard receiver operating characteristic (ROC) curve: the Feature-Response curve characterizes the sensitivity (coverage) of the sequence assembler output (contigs) as a function of its discrimination threshold (number of features/errors). Given a set of features, the response (quality) of the assembler output is then analyzed as a function of the maximum number of possible errors (features) allowed in the contigs. More specifically, for a fixed feature threshold 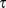, the contigs are sorted by size and, starting from the longest, only those contigs are tallied, if their sum of features is less than 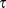. For this set of contigs, the corresponding approximate genome coverage is computed, leading to a single point of the Feature-Response curve.

Vezzi *et al.* [3] analyzed Feature space using multivariate techniques (*i.e.*, PCA and ICA) in order to study features’ interactions and to use these to select the most important ones. Such study, however, highlighted one of the main weak points of FRCurve: the need of a layout file, that is, a file describing the positions and orientations of each read (and therefore, each pair). While this file had been standard with old Sanger-based assemblers, only a small fraction of NGS-based assemblers provide such information (*i.e.*, Velvet, Ray, Sutta). Another relevant problem, deeply connected to the first, is the fact that features were computed by *amosvalidate*. Such features are commonly available for Sanger reads, clearly characterized by widely-varying insert-size distributions and expected coverages.

Results summarized in this paper clearly show that 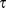 is able to effectively detect mis-assemblies and that it is able to rank assembler performances. The tool achieves high sensitivity and high specificity thus demonstrating that the implemented features are able to capture the large majority of the problems. Currently 9 features are computed using reads from paired-end libraries, while other 5 are computed using reads from a mate-pair library. FRCurve is computed using all of them, however the user is free to concentrate only on a subset of them (PCA can be used as shown in [3] to study features, see Document S1). New forensics features can be easily added to the program in order to highlight new problematic regions: small indels can be identified using reads aligned with gaps (*i.e.*, reads aligned with Smith-Waterman-like algorithm), problems in reconstructing gene space can be identified using RNA-seq reads, physical-maps or long single-molecule-sequences can be used to compute features, highlighting scaffolders’ performance.

Mapping reads back to the assembly provides only a rough approximation of the layout generation, especially in presence of repeat-structures: in such cases, reads that belong to correctly (or incorrectly) reconstructed duplicated regions can only be mapped randomly on one of the possible occurrences, thus, jeopardizing the hope of obtaining a correct layout. FRCurve’s ability to detect mis-assemblies is clearly limited by the presence of non-uniquely aligning reads (*i.e.*, reads aligning optimally in two or more positions). Thus, as the repetitive structures in a genome increase, which complicates the assembly problem, so does the difficulty in providing valid assembly evaluation. As the read-lengths increase or mate-pairs of different lengths become feasible, not only does the assembly problem become more tractable, but also new features enable better identification of problematic regions.

Despite the severe limitations imposed by the strategy of approximating read layout with read alignment, the present trend suggests that assemblers may continue to avoid producing layout files. Thus, it is believed that 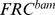 and, more in general, forensics features, will need to be computed by mapping reads back to the assembled sequence. The approach to approximate the layout by mapping reads back to the assembly has several advantages: (*i*) to potentially scale to any genome size (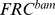 is currently being used to evaluate Spruce genome assembly, which will produce a reference genome of length 20 Gbp); (*ii*) to possibly compute new forensics features; (*iii*) and finally, to study relationships among features in a more uniform way.

Thanks to the feature-by-feature analysis, the FRCurve is often able to express and explain the current limitations of different assemblers. In many situations it is straight-forward to rank the assemblers simply by inspecting the FRC curves. Even when the scenario is unclear, FRCurve is still useful to highlight advantages and disadvantages of one assembler over the other (*e.g.*, an assembler that presents good long range connectivity but makes many mistakes in the small contigs, versus an assembler that has low connectivity but does not present local mis-assemblies). It is important to recall that, currently, none of the standard *de novo* evaluation metrics is able to capture these situations in the absence of a reference sequence.

We believe that features-based analysis will guide efforts aimed at *de novo* assembly evaluation and *de novo* assembler design. Our results clearly show that FRCurve can easily separate the best assemblies from the worst ones. By comparing feature-specific curves one can evaluate strong and weak points of each assembler and choose the system that best fits one’s objective. It is hoped that, in future, assembler-developers will be guided by the features-based analysis to improve these tools – at the core of the current genomic revolution.

## Software and Data Availability

The sequencing data used in this study is publicly available on the GAGE website and on the Assemblathon website (details are available in Document S1). 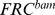 source code can be downloaded from https://github.com/vezzi/FRC_align.git.

## Supporting Information

Document S1 **Supplementary material.** This document contains the supplementary material and a detailed description to how reproduce results presented in the paper.
(PDF)
