# Supplementary material for: Reevaluating Assembly Evaluations with Feature Response Curves: GAGE and Assemblathons
Source: PLoS One. 2012 Dec 28;7(12):e52210. doi: 10.1371/journal.pone.0052210 (PMC3532452; doi:10.1371/journal.pone.0052210)
Supplement: Document S1 — Supplementary material. This document contains the supplementary material and a detailed description to how reproduce results presented in the paper. (PDF) [file pone.0052210.s001.pdf]

Reevaluating Assembly Evaluations using Feature Analysis: GAGE and Assemblathons
Supplementary material

Francesco Vezzi, Giuseppe Narzisi, Bud Mishra

September 29, 2012

Features computation

FRC^{bam} computes features using a sliding window of size W. By default W is set to 1 Kbp, and in each step it slides by 200 bp. Let A denote a genome to be assembled (*i.e.*, in other words it is the desired output). Let $\mathcal{R} = \{r_1^1, r_1^2, \ldots, r_n^1, r_n^2\}$ denote the set of sequenced paired reads from A. Pairs are at a known estimated distance, d (and standard variation, v) and with known orientations. FRC^{bam} input is:

- $\mathcal{A} = \{a_1, \ldots, a_m\}$ denotes the set of contigs produced when assembled from $\mathcal{R}$;
- S denotes the alignment of $\mathcal{R}$ against $\mathcal{A}$.

Every read $r \in \mathcal{R}$ has a record in S, $S[r]$, describing the alignment of a read. If a read can be aligned in more than one position then only one of these alignments is stored in S. For each entry in S we define the following fields:

- $S[r].seq =$ the sequenced read r itself;
- $S[r].c =$ contig where read aligns, empty if read has no alignment;
- $S[r].p =$ alignment start position on $S[r].c$, -1 if read has no alignment;
- $S[r].s =$ strand orientation (*for* [forward] or *rev* [reverse]);
- $S[r].m =$ mated read;
- $S[r].ins =$ insert length (computed as the distance between the beginning of the alignment of the leftmost read and the end of the alignment of the rightmost read)

A pair (r_i^1, r_i^2) is said to be *properly aligned* on $\mathcal{A}$ if r_i^1 and r_i^2 align on the same contig $a_j \in \mathcal{A}$ and are at the expected distance and orientation. More precisely, (r_i^1, r_i^2) with $S[r^1].p < S[r^2].p$ and with minimum and maximum expected distance respectively m and M properly aligns iff:

$$S[r_i^1].c = S[r_i^2].c \ \wedge \ S[r_i^1].s = \mathit{for} \ \wedge \ S[r_i^2].s = \mathit{rev}$$

$$\wedge \ m \leq S[r_i^1].ins \leq M.$$

The condition applies similarly when $S[r_i^1].p \geq S[r_i^2].p$.

Let us define the function $\mathit{pair}(r)$ to return the read paired to r stored in $\mathcal{R}$ and the function $\mathit{proper}(S[r])$ to return the insert size between the read r and its mate if they form a proper pair and -1 otherwise.

Once reads are aligned we can extract (at least) two kinds of coverages: *read coverage* and *span coverage*. Given a region I and a set of reads $\mathcal{R}'$ that align on I the read coverage is:

$$C = \frac{\sum_{r \in \mathcal{R}'} |r|}{|I|}.$$

Similarly, given a region I and a set of paired reads $\mathcal{R}'$ all properly aligned on I, the span coverage is defined as

$$S = \frac{\sum_{(r^1,r^2) \in \mathcal{R}'} \mathit{proper}(S[r^1])}{2|I|}.$$

In the case of the span coverage the dividing factor 2 is necessary in order to avoid double-counting each proper pair.

The alignment input file S can be used to further divide $\mathcal{R}$ into subsets:

- $\mathcal{R}_A = \{r_A \ : r_A \in \mathcal{R} \ \wedge \ S[r_A].p \geq 0\}$: the set of all reads that align on the assembly $\mathcal{A}$;
- $\mathcal{R}_M = \{r_M \ : r_M \in \mathcal{R} \ \wedge \ \mathit{proper}(S[r_M]) \geq 0\}$: reads that align with the pair at the correct distance and orientation;
- $\mathcal{R}_W = \{r_W \ : r_W \in \mathcal{R} \ \wedge \ S[r_W].p \geq 0 \wedge S[\mathit{pair}(r_W)].p \geq 0 \wedge S[r_W].c = S[\mathit{pair}(r_W)].c \wedge \mathit{proper}(S[r_W]) = -1\}$: reads that align with the pair on the same contig but at a wrong distance and/or orientation;
- $\mathcal{R}_S = \{r_S \ : r_S \in \mathcal{R} \ \wedge \ S[r_S].p \geq 0 \wedge S[\mathit{pair}(r_S)].p = -1\}$: singleton reads;
- $\mathcal{R}_C = \{r_C \ : s_{r_S} \in \mathcal{R} \ \wedge \ S[r_S].p \geq 0 \wedge S[\mathit{pair}(r_S)].p = -1 \wedge S[r_S].c \neq S[\mathit{pair}(r_S)].c\}$: paired read properly placed but in another contig.

Given $\mathcal{A}$, $\mathcal{R}$ and S we can compute the total read coverage C_A and the total span coverage S_A :

$$C_A = \frac{\sum_{r_A \in \mathcal{R}_A} |r_A|}{|\mathcal{A}|}; \qquad S_A = S_M = \frac{\sum_{r \in \mathcal{R}_M} |\mathit{proper}(S[r])|}{2|\mathcal{A}|}.$$

In an almost identical way we can compute also:

- C_M read coverage induced by correctly aligned pairs on the overall assembly $\mathcal{A}$;
- C_W read coverage induced by wrongly mated reads;
- C_S read coverage induced by single reads;
- C_C read coverage induced by reads with mate on different contigs.

Given $\mathcal{A}$, $\mathcal{R}$ and S we can also compute a local coverage. Let us define W the *window size*, then for a given contig $a \in \mathcal{A}$ and a given coordinate $i \in \{0, \ldots, |a| - W - 1\}$ we can identify the set of reads that align on the selected contig between i and $i + W$:

$$\mathcal{R}_A^{a,i} = \{r \in \mathcal{R}_A : S[r].c = a \wedge S[r].p \in \{i, \ldots, i + W - 1\}\}.$$

Again, in a similar way we can compute $\mathcal{R}_M^{a,i}$, $\mathcal{R}_W^{a,i}$, $\mathcal{R}_S^{a,i}$, and $\mathcal{R}_C^{a,i}$.

The local read and span coverages with respect to the window size W at contig a in position i are defined as:

$$C_A^{a,i} = \frac{\sum_{r \in \mathcal{R}_A^i} |r|}{W}; \qquad S_A^{a,i} = \frac{\sum_{r \in \mathcal{R}_M^{a,i}} |\mathit{proper}(S[r])|}{2W}.$$

Also in this case we can easily define and compute $C_M^{a,i}$, $C_W^{a,i}$, $C_S^{a,i}$, and $C_C^{a,i}$.

Compression Expansion statistics

Compression/Expansion (CE) statisistics detect misassemblies of compressions or expansions. Such misassemblies are usually a consequence of the difficulties in (correctly) assembling repeated regions.

For a given location in a draft assembly, we examine the sample of inserts from a given library that span this location in the genome. Using the read placement coordinates from the assembly, we compute the mean M of the implied insert lengths l_i for the sample. More precisely, if N is the local coverage, or the number of inserts in a sample, and the lengths are denoted $l_1, \ldots, l_N$ then:

$$M = \frac{1}{N} \sum_{i=1}^{N} l_i.$$

The CE statistic is based on the fact that the variance of the sum of independent random variables is equal to the sum of their variances. The statistic measures the distance between the mean M of the local sample and the library mean μ in the units of expected sample standard deviation $\sigma/\sqrt{N}$. We define the value of the CE statistic Z as

$$Z = \frac{M - \mu}{\sigma/\sqrt{N}}.$$

For a given contig $a \in \mathcal{A}$ we can compute for each non-overlapping window of size W the CE statistic of such area $Z^{a,i}$ and check if it is between CE_{min} and CE_{max}. If it fails, then such windows will be marked as `CE_stretch` or `CE_compr`.

CE_{min} and CE_{max} estimation

An obvious problem with CE statistics is the threshold estimation to be used in detecting misassemblies, while avoiding both false positives and negatives. As noted by Zimin *et al.* [11], insert sizes are distributed approximately normally, with the local means also being approximately normally distributed. Hence in the perfect assembly, with the availability of the correct layout, the number of locations $N(T)$ for which the CE statistic is above T or below $-T$ must correspond to what would occur for a normal distribution. Following Zimin *et al.* approach we plotted, for each dataset and for each assembly the number of locations N as a function of the threshold T for each library. For $T > 0$ we count all events in which the CE statistic value was greater than T, and for $T < 0$ we count all events in which the CE statistic value was lower than T. We then plot the Gaussian fit in order to capture the Gaussian part of all tested sequences.

Thanks to the availability of the reference sequence for four of our five datasets (the three GAGE datasets and Assemblathon 1) we could also plot the same curve using directly the mappings to the reference. This way we can estimate the noise level introduced by approximating the layout with the alignment, and also check if our threshold estimation is correct.

Staphilococus

Without using the available reference plot (thick blue line), we opted for the following thresholds: $(-4, 4)$ and $(-3, 5)$ for pair read and mate pairs respectively.

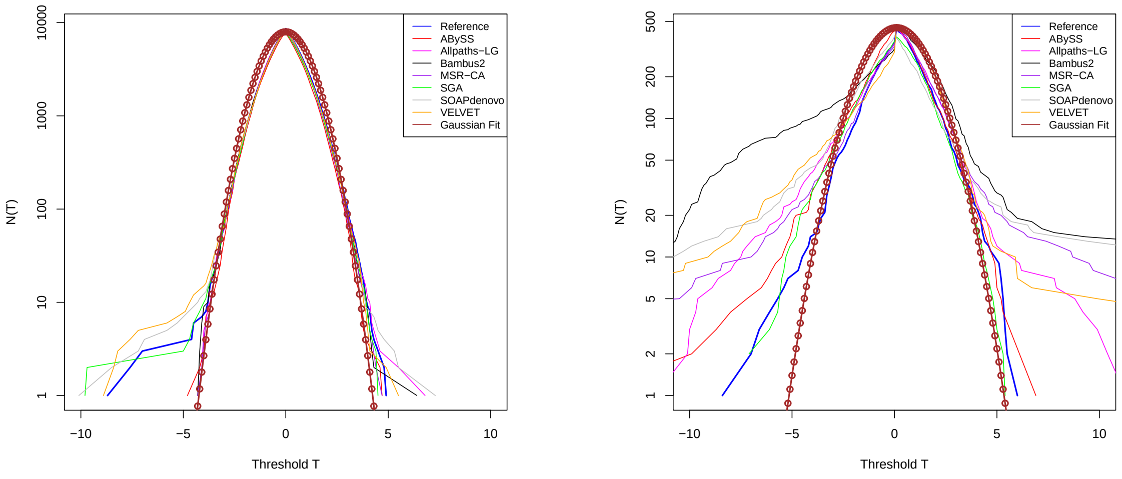

(a) CE statistics distribution on pair ends.

(b) CE statistics distribution on mate pair.

Figure 1: CE statistics computed on paired end library for Staphylococcus dataset. Distribution of the number of location where CE statistics are higher than T for $T > 0$ and lower than T for $T < 0$.

Rhodobacter

Without using the available reference plot (thick blue line), we opted for the following thresholds: $(-5, 5)$ and $(-6, 7)$ for pair read and mate pairs respectively.

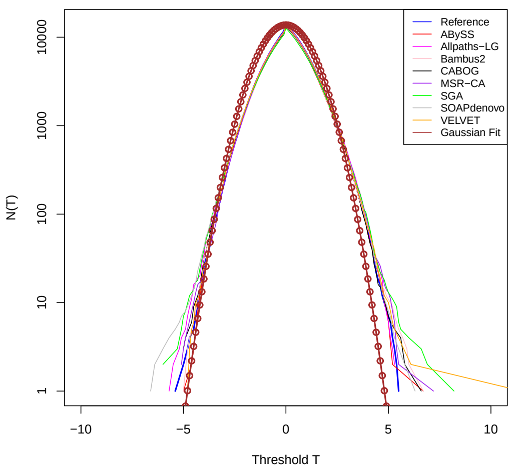

(a) CE statistics distribution on pair ends.

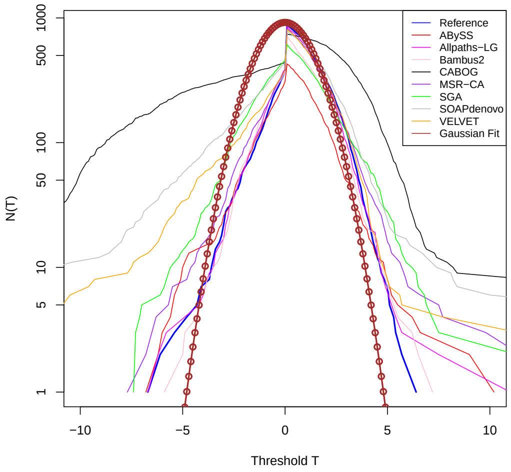

(b) CE statistics distribution on mate pair.

Figure 2: CE statistics computed on paired end library for Rhodobacter dataset. Distribution of the number of location where CE statistics are higher than T for $T > 0$ and lower than T for $T < 0$.

Human Chromosome 14

Without using the available reference plot (thick blue line), we opted for the following thresholds: $(-9, 10)$ and $(-14, 14)$ for pair read and mate pairs, respectively. In the mate pair case (see Figure 3(b)) we plotted two Gaussian-fit curves, as it was clearly more difficult to approximate the distribution of the various assemblies. This distribution is likely to be a consequence of the simulation process used in generating such datasets.

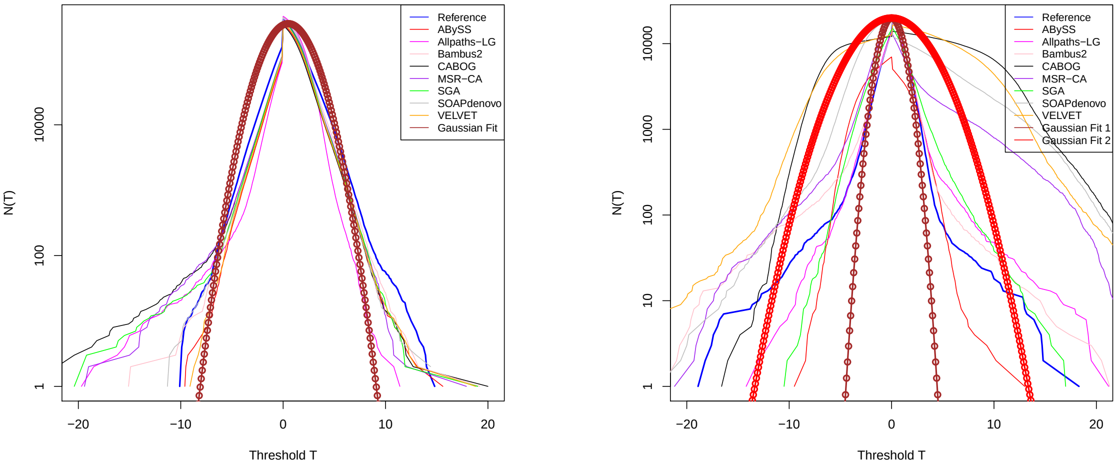

(a) CE statistics distribution on pair ends.

(b) CE statistics distribution on mate pair.

Figure 3: CE statistics computed on paired end library for Human Chromosome 14 dataset. Distribution of the number of location where CE statistics are higher than T for $T > 0$ and lower than T for $T < 0$.

Assemblathon 1

Without using the available reference plot (thick blue line), we opted for the following thresholds: $(-4, 4)$ and $(-5, 5)$ for pair read and mate pairs respectively.

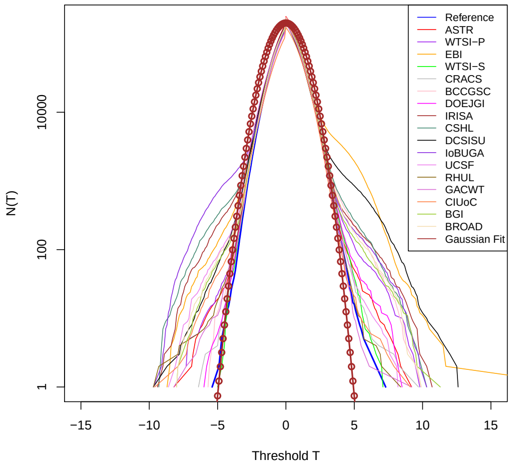

(a) CE statistics distribution on pair ends.

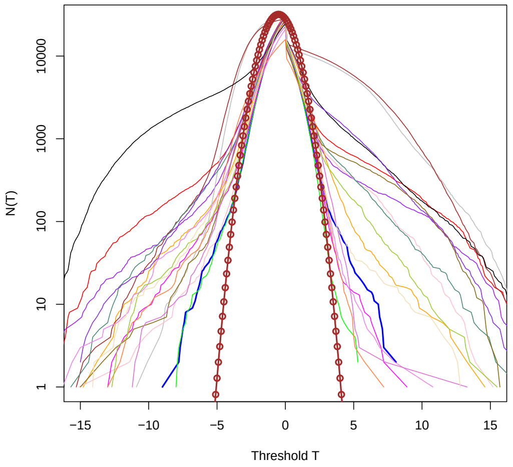

(b) CE statistics distribution on mate pair.

Figure 4: CE statistics computed on paired end library for Assemblathon 1 dataset. Distribution of the number of location where CE statistics are higher than T for $T > 0$ and lower than T for $T < 0$.

Assemblathon 2

Assemblathon 2 dataset has no a reference sequence. By plotting the Gaussian Fit (thick blue line) we opted for the following thresholds: $(-6, 6)$ and $(-5, 5)$ for pair read and mate pairs respectively.

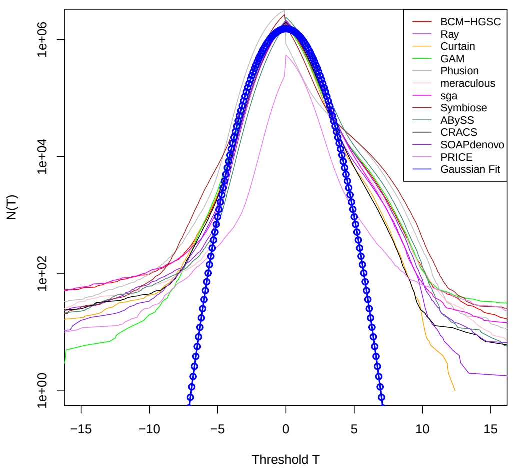

(a) CE statistics distribution on pair ends.

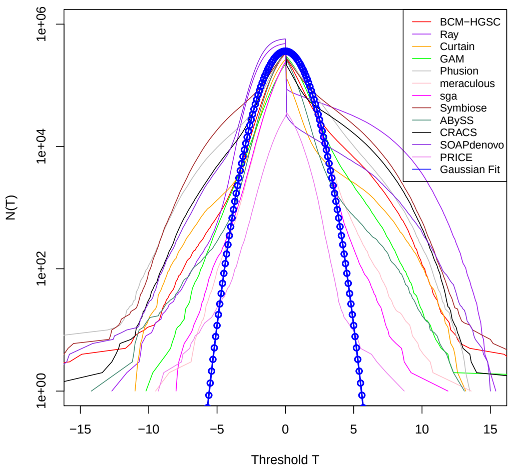

(b) CE statistics distribution on mate pair.

Figure 5: CE statistics computed on paired end library for Assemblathon 2 dataset. Distribution of the number of location where CE statistics are higher than T for $T > 0$ and lower than T for $T < 0$.

Feature computation

In order to compute features we used a sliding window approach. The default window size is 1Kbp and it slides in steps of 200bp. The intention is to extend feature areas as far as possible. However, the number of features is always computed as a function of the window size, thus avoiding the problem that assembly A may have a lower number of features than assembly B, but that the total length of the areas affected by features in A is larger than B. Below, we present the pseudo code for the computation of one feature (*i.e.*, `LOW_COV`):

Algorithm 1: COMPUTE LOW_COV

Input: $\mathcal{A}$ *de novo* contig set;
Output:

procedure ComputeLowCov(S)
  C_A;
  $FEATURES \leftarrow 0$
  foreach $a \in \mathcal{A}$ **do**
    $feature \leftarrow$ FALSE; $feat\_Start \leftarrow 0; feat\_End \leftarrow 0$;
    compute $C_A^{a,0}$
    if $C_A^{a,0} < \frac{1}{2}C_A$ **then**
      $feature \leftarrow$ TRUE; $feat\_Start \leftarrow 0$; $feat\_End \leftarrow W$;
    $i = 0$;
    while $i < |a|$ **do**
      compute $C_A^{a,i}$;
      if $C_A^{a,i} < \frac{1}{2}C_A$ **then** (`//check if there is a feature`
        if feature **then** `//check if this is an extension`
          $feat\_End \leftarrow feat\_End + 200$;
        else `//or if it is a new one`
          $feature \leftarrow$ TRUE; $feat\_Start \leftarrow i$; $feat\_End \leftarrow i + W$
      else `// no feature found in this window`
        if feature **then** `//if before there was a feature close it`
          $FEATURES \leftarrow FEATURES + round\left(\frac{feat\_End - feat\_Start}{W}\right)$;
          $feature \leftarrow$ FALSE; $i \leftarrow feat\_End$
      $i \leftarrow i + 200; feat\_End \leftarrow i + 200$
  return $FEATURES$;

Multiple Library Features

Often the assembly process involves more than one library. For Illumina we typically have at least two libraries: Paired End library (PE), and Mate Pair library (MP). The former are paired reads in the standard orientation ($\rightarrow \leftarrow$) with insert size that can vary between 180 bp (overlapping fragments) and 600 bp (standard PE). The latter are sequenced in the opposite direction ($\leftarrow \rightarrow$) and the insert size is much longer (usually between 3 and 10 Kbp).

As a consequence of the different protocols and costs usually a project is designed with a high coverage of PE reads but a more modest MP read coverage. The main advantage of MP reads is to improve contiguity through scaffolding and gap-filling procedures.

The current implementation of FRC^{bam} accepts at most 2 libraries: one mandatory PE library and another optional MP library. The user needs to provide a rough estimation of the insert size and of the standard deviation, although this information is used only to discard outliers. Commonly, both insert size and standard deviation are recomputed based on the alignments (MP should be reversed and complemented prior to aligning).

These two libraries are used in turn to compute the following 14 features: 9 PE-based features and others 5 PE-based features. The 9 PE-based features are:

- `LOW_COV_PE`: areas characterized by *low read coverage* (*i.e.*, by default two times lower than the average read coverage computed from the alignment);
- `HIGH_COV_PE`: areas characterized by *high read coverage* (*i.e.*, by default two times higher than the average read coverage computed from the alignment)
- `LOW_NORM_COV_PE`: areas characterized by *low paired-read coverage* (*i.e.*, coverage computed only on correctly aligned paired reads, same default value as `LOW_COV_PE`);
- `HIGH_NORM_COV_PE`: areas characterized by *high paired-read coverage* (*i.e.*, coverage computed only on correctly aligned paired reads, same default value as `HIGH_COV_PE`);
- `COMPR_PE`: areas characterized by *low CE-statistics* (*i.e.*, default value set to -3, see CE_{min} and CE_{max} estimation section for more details);
- `STRECH_PE`: areas characterized by *high CE-statistics* (*i.e.*, default value set to 3, see CE_{min} and CE_{max} estimation section for more details);
- `HIGH_SINGLE_PE`: areas characterized by *high number of reads with unmapped pair* (*i.e.*, by default 0.4 of the read coverage is composed by reads with unplaced pair);
- `HIGH_SPAN_PE`: areas characterized by *high number of reads with pair mapped in a different contig/scaffold* (*i.e.*, by default 0.4 of the read coverage is composed by reads with pair mapped in a different contig/scaffold);
- `HIGH_OUTIE_PE`: areas characterized by *high number of mis-oriented or too distant paired reads* (*i.e.*, by default 0.4 of the read coverage is composed by mis-oriented reads);

while the 5 MP-based features are::

- `COMPR_MP`: areas characterized by *low CE-statistics* (*i.e.*, default value set to -5, see CE_{min} and CE_{max} estimation section for more details);
- `STRECH_MP`: areas characterized by *high CE-statistics* (*i.e.*, default value set to 3, see CE_{min} and CE_{max} estimation section for more details)
- `HIGH_SINGLE_MP`: areas characterized by *high number of reads with unmapped pair* (*i.e.*, by default 0.4 of the read coverage is composed by reads with unplaced pair and read coverage must be at least 2);
- `HIGH_SPAN_MP`: areas characterized by *high number of reads with mate mapped in a different contig/scaffold* (*i.e.*, by default 0.4 of the read coverage is composed by reads with mate mapped in a different contig/scaffold and coverage should be at least two);
- `HIGH_OUTIE_MP`: areas characterized by *high number of mis-oriented or too distant paired reads* (*i.e.*, by default 0.4 of the read coverage is composed by mis-oriented reads and coverage should be at least two).

Coverage based features are likely to identify copy number variation problems: an area characterized by a high coverage is likely to indicate a failure of the assembler in guessing the right number of copies of a sequence. CE-based features are able to identify insertion deletion events. Different insert sizes can be used to infer different types of events. `HIGH_SINGLE` features identifies areas that are likely to be missing from the assembly: if several reads are aligned without their pair/mate a likely explanation is that the assembler failed in assembling a genome locus. In other words, an area marked as `HIGH_SINGLE_PE` and `HIGH_SINGLE_MP` indicates a possible assembler failure in a nearby area. `HIGH_SPAN` features are indicative of areas characterized by a large number of spanning reads suggesting either a scaffolder failure in connecting contigs, or scaffolding errors. Lastly, `HIGH_OUTIE` features identify areas characterized by a large number of mis-oriented pairs/mates, thus indicating possibly rearranged segments of the assembly.

Usually, PE reads provide a high read coverage (*i.e.*, vertical coverage) but a low spanning coverage (*i.e.*, horizontal), while MP reads provide a low read coverage but produce a high spanning coverage. PE reads are usually evenly sequenced along the genome, while MP libraries are often subject to sampling and redundancy problems (*e.g.*, the library does not represent all the genome, or some genomes areas are over-sequenced). For these reasons MP reads are used only to compute CE-statistics in order to identify insertion deletion events, and to highlight regions composed by a large number of singleton, spanning, or wrongly oriented reads.

Read Alignment Limitations

Approximating the assembly layout-file with read alignment is, obviously, an error-prone approximation. Reads may align in wrong locations as a consequence of sequencing errors, highly similar or perfect repeats, and collapsed/expanded heterozygous loci. Usually, in presence of repeats, reads are assigned a random position among the possible alignments. Coverage based features are computed on all aligned reads, while paired/mate based features (*e.g.* CE-statistics features) are computed only on paired reads such that at least one pair can be aligned in a single position (*i.e.*, the multiple occurring read in the pair will be possibly anchored in the most likely place according to the insert size).

This section will explore some of the limits of approximating the read layout with read alignment. In particular, we will estimate the noise introduced by the alignments (*i.e.*, false positives), the ability to detect features in presence of copy number variation events, and how different alignment strategy can improve the currently adopted strategy.

In order to evaluate the limits of approximating the reads' layout, we aligned against *Staphylococcus a.* reference sequence the same reads used to assemble this genome in the GAGE project [7]. We identified 25 different features. The vast majority of them were approximately 1Kbp long (*i.e.* the minimum length) and covered a total of 0.81% of Staphylococcus genome. These features have to be treated as the noise introduced by the alignment (however they can also be a consequence of sequencing bias). The longest area marked as a feature was a 5 Kbp area flagged as `LOW_NORM_COV_PE`. By aligning this 5Kbp sequence against the reference itself we discovered that it was a repetitive sequence, and the vast majority of its reads were aligned as multiple (and hence there were no proper paired reads associated with it).

This experiment confirmed that the most problematic regions are those in which short reads align in multiple copies (*i.e.*, ambiguous reads). As a matter of fact, aligners return only one possible alignment for each ambiguous read (they pick up one random alignment or, if the pair/mate aligns uniquely the alignment that respect the insert size) and they flagged it as multiple (*e.g.*, BWA gives to such alignment 0 quality). We tried to assess how our features, in particular the coverage based ones, are affected by the presence of ambiguous reads. To reach our goal we computed features using ambiguous reads in three different ways:

1. **stringent**: discard reads aligned in multiple positions;
2. **default**: choose a random alignment;
3. **total**: keep track of all possible alignments.

We first created an *in vitro* Staphylococcus genome sequence, by inserting in 9 different non overlapping locations a perfect 10Kbp sequence denoted A. Let us call this new reference sequence S^{A10}. Note that A is

present in single copy in the original Staphylococcus sequence and 2380 out of 2417 reads aligning on A in Staphylococcus genome are uniquely mapped. We created a new set of reads $\mathcal{R}^{10}$ composed of the original Staphylococcus reads (GAGE datasets, refer to [7]) plus 10 copies for each of the 2380 uniquely aligned reads over R. In other words, we created a new reference sequence and the set of reads sequenced from it. We then created 9 *in vitro* sequences (*i.e.*, S^{A1},S^{A2},...,S^{A9}) with sequence S^{Ai} containing i copies of A. At this point we aligned $\mathcal{R}^{10}$ against S^{A1},S^{A2},...,S^{A9},S^{A10} and computed features using alignments. In this situation, S^{A10} represents the correct sequence, while all the other "assemblies" have failed in reconstructing A with the correct copy number.

Reads have been aligned using the **default** strategy (*i.e.*, only random hit is reported for each ambiguous read), and the **total** strategy (*i.e.*, report all alignments). We then computed `LOW_COV` and `HIGH_COV` using the **stringent**, **default**, and the **total** strategy .

The **stringent** and the **total** strategy are extremely similar. When ambiguous reads are not used to compute features all areas composed by repetitive elements are ignored (they form a sort of genomic dark matter [2]). Such areas can be considered too difficult to analyse. As an example consider S^{A9}: any method that is not based on a reference sequence will find it extremely difficult to determine that a copy is missing. When this strategy is adopted on the real Staphylococcus reference only 6 features are identified, 3 of them at the start and at the end of the genome, which is simply a consequence of its circularity.

When all hits are reported, all areas in S^{A1},S^{A2},...,S^{A10}, where A has been inserted are categorized as `HIGH_COV`. However this information does not tell much more, as compared to just ignoring multiple alignments. One has to find a way to estimate the correct copy number of the repeat and, without the presence of a reference sequence, this problem may not suggest any single "correct solution."

When only one random hit is reported for each ambiguous read (*i.e.*, **default** strategy) coverage-based features (*i.e*, `HIGH_COV` features) flagged with high precision all areas in S^{A1},S^{A2},S^{A3}, and S^{A4} where a copy of A was inserted (*i.e.*, the features were able to identify the copy number variation problem). In S^{A5} all the 5 copies of A were partially labelled by coverage-based features, while S^{A6} and the others did not show any coverage based feature in presence of the repeated sequence. This was clearly expected as the high coverage cutoff is set to 2 (all position with coverage higher than twice the average coverage are marked as `HIGH_COV`). Lowering this threshold is a possible solution, however more false positives are likely to be introduced. It must be noticed, however, that other features like `HIGH_OUTIE` and `COMPR` can be used to identify the presence of assemblies that contains sequences assembled in wrong copy numbers.

Approximating assembly layout with read alignment is, to the best of our knowledge, the only way to compute features and evaluate assemblies. This experiment demonstrates, on the one hand, a limit of this approach (*i.e.*, wrong copy number estimation of a highly repeated sequence), but, on the other hand, showed that the **default** alignment strategy is able to easily identify large copy number problems (*e.g.*, using coverage based features) and to raise suspicions about less evident copy number problems (*e.g.*, using CE or long range based features).

FRC evaluation

Sensitivity and Specificity

In order evaluate the ability of features to correctly identify mis-assemblies we used *dnadiff* output. With access to a reference sequence we can align de novo contigs against it and infer all the real mis-assemblies. This approach allows a user to evaluate in a quantitative way the amount of mis-assemblies produced by each assembler and understand how realistic the FRCurve and the features are. Moreover, by inspecting the alignments of the contigs to the reference, we compute feature's sensitivity and specificity according to the following equations:

$$Sensitivity = \frac{True\ Positives}{True\ Positives + False\ Negatives};$$

$$Specificity = \frac{True\ Negatives}{True\ Negatives + False\ Positives}.$$

Figure 6 shows how to use the real mis-assembly areas to compute predictiveness of each feature.

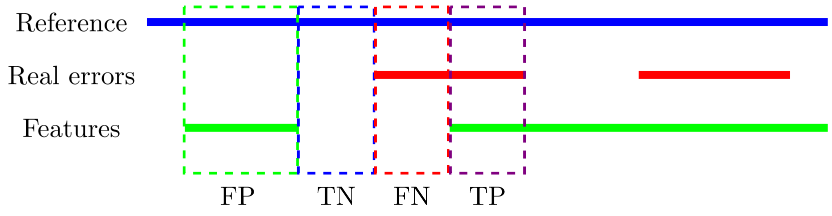

Figure 6: Computation of TP, TN, FP, and FN areas

FRC^{amos} vs FRC^{bam}

FRCurve can be computed using *amosvalidate* and it is already a part of `amos` package. However, because of the paucity of tools capable of returning as output an `afg` file or bank file, it limits the application of FRC tools. In order to overcome these limitations, we implemented a new procedure for computing features from an alignment file (`bam`). Features computed from `amos` (*i.e.*, computed with FRC^{amos}) and features computed from an alignment (*i.e.*, features computed with FRC^{bam}) are inherently different, nonetheless we expect them to be significantly correlated. Moreover FRCurves obtained from FRC^{amos} and FRC^{bam} are also presumed to be similar. Features computed by FRC^{amos} were characterized by a high sensitivity, although they were affected by a low specificity, especially when applied to NGS datasets [9].

In order to verify these premises we computed features and FRCurves of different assemblers that generate the layout file and compare it with FRC computed from alignment. To the best of our knowledge only the following NGS assemblers return a layout file that *may* be processed by *amosvalidate*: Velvet [10], Ray [1], CABOG [4], and Sutta [5]. Velvet produces an *afg* file that can easily be converted in a *bank* file and processed with *amosvalidate*. Also Ray produces an afg file that, however, is incomplete (missing library description and paired end information). We had to complete it "manually" in order to run *amosvaliadate*. We were not able to use either GABOG or Sutta. Even though CABOG output should be easy to transform into afg format, we were not able to perform this action. This problem could probably be attributed to an incomplete integration of new and old formats (probably the program that converts illumina reads in CABOG input format outputs data in a format ill-suited for *toAmos* program). SUTTA is based on amos pipeline and therefore its output can easily be processed with amos tools. However, the publicly available implementation works only with ultra-short Illumina reads (*i.e.*, shorter than 50).

We therefore decided to compute FRCurves using Ray and Velvet on *Staphylococcus aureus* dataset from GAGE. We computed amos-based features (*i.e.*, FRC^{amos}) and alignment based features (*i.e.*, FRC^{bam}). Moreover we used *dnadiff* [6] to localize real mis-assemblies (same strategy used by GAGE).

Figure 7 shows the FRCurves computed with amosvalidate and with FRC^{bam}. In order to compute FRC with Amos we allowed only the computation of comparable features, so we discarded SNP-based features.

We can see how similar Figures 7(a) and 7(b) are. The relationship between the two assemblies is quite close and also the shapes of FRC are similar, which suggests that features produced by FRC^{bam} are just as predictive as features obtained through FRC^{amos}, although the absolute number of features computed by FRC^{amos} is much higher than the ones computed by FRC^{bam} (see Fig. 7). In order to understand this difference we checked the different features found by FRC^{amos} and FRC^{bam}: the most represented feature is always connected to the presence of areas made of reads without the mate (`HIGH_SINGLEMATE_CVG` for FRC^{amos}, and `HIGH_SINGLE_MP` for FRC^{bam}). FRC^{amos} and FRC^{bam} identify 39,830 and 1205 of such features respectively. Also the second most frequent features are closely related: `HIGH_LINKING_CVG` and `HIGH_SPAN_MP` are both suggestive of failures in the scaffolding phase. One of the possible explanations of the large difference in numbers is the sliding window approach implemented by FRC^{bam}. However, another likely explanation, supported by the results shown in Table 1, is that FRC^{bam} are able to capture NGS

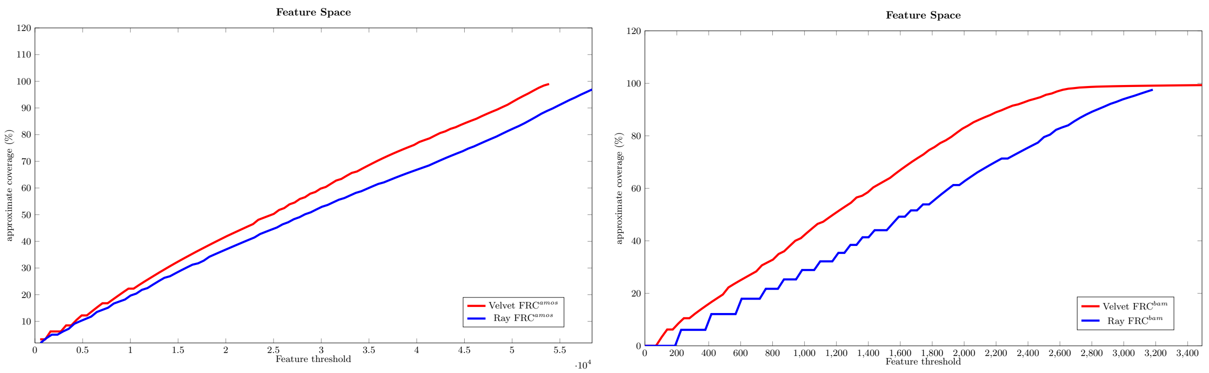

(a) FRCurve computed with FRC^{amos}

(b) FRCurve computed with FRC^{bam}

Figure 7: Staphylococcus GAGE dataset

features in a more sensible way.

	# Ctg	N50 (Kbp)	ERRORS				AMOS		BAM		covered genome	
			inser	trans	Inv	% Real Errors	sens	spec	sens	spec	% AMOS feat	% BAM feat
Velvet	303	21.5	106	1	6	2.5%	0.91	0.36	0.93	0.86	75.7%	23%
Ray	438	10.5	189	10	1	3.4%	0.99	0.22	0.90	0.89	78.0%	18.4%

Table 1: Comparison between Velvet and Ray amos/based features and alignment based features.

Table 1 summarizes the analysis performed with *dnadiff* in order to evaluate assemblies using the original reference sequence. Number of contigs and N50 are not really informative for our purpose, however they show Velvet producing more contiguous contigs. Ray produces a larger set of smaller contigs. Concerning mis-assembly counting, we can see that Velvet is less affected by translocation and inversion events. However, the difference in absolute numbers is not large. It is clear that Table 1 is in agreement with the FRCurves in Figure 7.

We may now carefully examine next three sections of Table 1. We computed sensitivity and specificity of FRC^{amos} and FRC^{bam} features respectively. As expected, FRC^{amos} features show a high sensitivity but a poor specificity, in agreement with the previous studies [6, 9]. Features computed on alignment still show a high sensitivity (despite lower than Amos-based features) but they have a much higher specificity. Features computed by FRC^{amos} cover more than 75% of Velvet assembly while Features computed by FRC^{bam} cover "only" 25% of the assembly: note that this reduction greatly improves specificity without affecting sensitivity.

Aligners

FRC^{bam} computes an approximate layout from an alignment in SAM/BAM format. It is clear that the choices performed by the aligner are essential in order to produce accurate features. We choose to use rNA [8] a short string aligner able to align reads in a highly sensitive way. We also tested FRC^{bam} with BWA [3], with the results summarized in Figure 8.

FRCurves generated from rNA alignment (*i.e.*, Figure 8(a)) and from BWA alignment (*i.e.*, Figure 8(b)) differ somewhat, however, the relationships among the different assemblers are almost the same. Only Velvet and SOAPdenovo appear to have switched ranks, although the absolute number of features is so close that it is hard to tell which is the better of the two.

Datasets

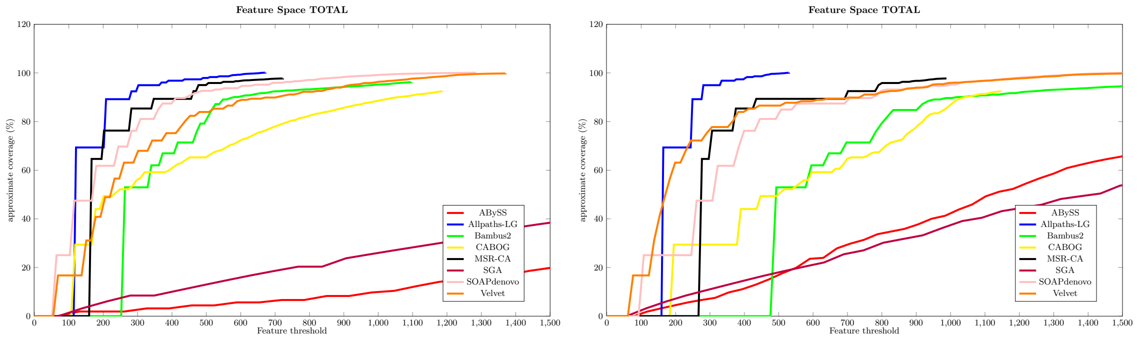

(a) FRCurves for Rhodobacter with rNA.

(b) FRCurves for Rhodobacter with BWA.

Figure 8: FRCurves on Rhodobacter scaffolds computed from an alignment obtained with rNA (Figure 8(a) and with BWA 8(b)

Dataset	Insert	location
Staphylococcus paired ends	180bp	`http://gage.cbcb.umd.edu/data/` `... Staphylococcus_aureus/Data.quakeCor.tgz`
Staphylococcus mate pairs	3500bp	`http://gage.cbcb.umd.edu/data/` `... Staphylococcus_aureus/Data.quakeCor.tgz`
Rhodobacter paired ends	180bp	`http://gage.cbcb.umd.edu/data/` `... Rhodobacter_sphaeroides/Data.quakeCor.tgz`
Rhodobacter mate pairs	3500bp	`http://gage.cbcb.umd.edu/data/` `... Rhodobacter_sphaeroides/Data.quakeCor.tgz`
Hc14 paired ends	155bp	`http://gage.cbcb.umd.edu/data/` `... Hg_chr14/Data.quakeCor.tgz`
Hc14 mate pairs	6283	`http://gage.cbcb.umd.edu/data/` `... Hg_chr14/Data.quakeCor.tgz`
Assemblathon 1 paired ends	300bp	`http://korflab.ucdavis.edu/Datasets/Assemblathon/` `Assemblathon1/speciesA_300i_40x.[12].fastq.bz2`
Assemblathon 1 mate pairs	3000bp	`http://korflab.ucdavis.edu/Datasets/Assemblathon/` `... Assemblathon1/speciesA_3000i_20x_r3.[12].fastq.bz2`
Assemblathon 2 paired ends	400bp	`http://bioshare.bioinformatics.ucdavis.edu/Data/hcbxz0i7kg/Snake` `/short_inserts/110210_EAS56_0249_FC62W0CAAXX_lane[1234567].fastq.gz`
Assemblathon 2 mate pairs	2000bp	`http://bioshare.bioinformatics.ucdavis.edu/Data/hcbxz0i7kg/` `Snake/mate_pairs/110405_EAS192_0222_FC70N35AAXX_lane[123].fastq.gz`

Table 2: Complete path to datasets used for the experiments.

More FRCurves and DotPlot validation

GAGE results comprise both scaffolds and contigs produced by the assemblers. In the main paper we focus on the final assemblies (*i.e.*, the scaffolded once). Here we present FRCurves for both contigs and scaffolds for each GAGE dataset: Staphylococcus see Figure 9, Rhodobacter see Figure 12, and Hc14 see Figure 15.

Moreover, for each dataset (*i.e.*, GAGE and Assemblathon 1 and 2), we plot the FRCurve for each distinct feature for each final assembly (See Figures 10, 13, 16, 19, and 21). Such FRCurves highlight behavior of any specific assembler. As an example consider Assemblathon 1 dataset: Broad Institute's Allpaths entry, in contradiction with the Assmeblathon1 paper analysis, is not one of the best performing assemblers. This disagreement is due primarily to the presence of two highly correlated features: `LOW_NORM_COV_PE` and `HIGH_SPAN_PE`. These two features occurred both in long and short contigs. In all the tested cases we discovered that this particular situation was a consequence of the presence of small contigs that perfectly align within some long contig, probably the result of an unresolved allele splitting. This fact is confirmed by the fact that Broad's Allpaths is only the 11 *th* ranking team in the Assemblathon 1 paper evaluation, based solely on copy number variations.

For each GAGE dataset we aligned the longest generated scaffold from each assembler against the reference sequence in order to partially confirm the FRCurves' shapes (see Figures 11, 14, and 17).

Staphilococus

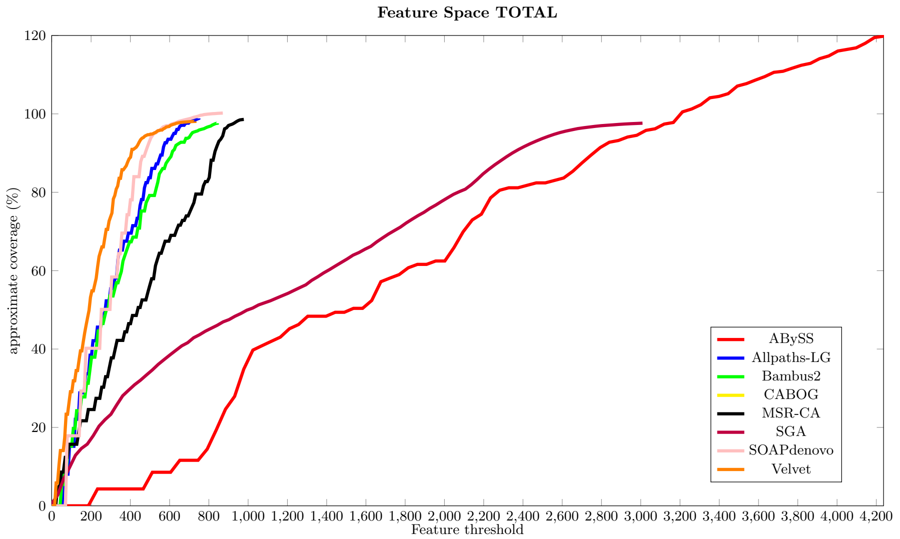

(a) FRCurves for Staphylococcus contigs.

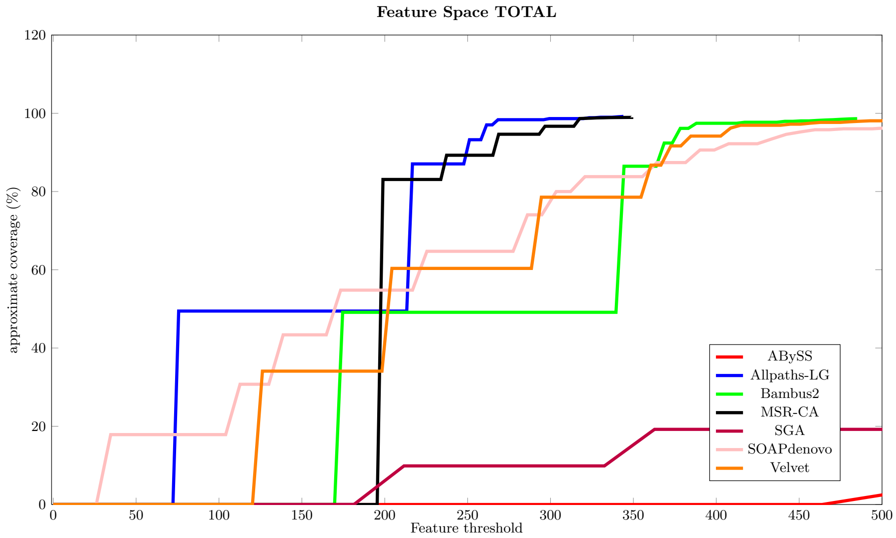

(b) FRCurves for Staphylococcus scaffolds.

Figure 9: FRCurves on Staphilococcus contigs and scaffolds assemblies available on GAGE website.

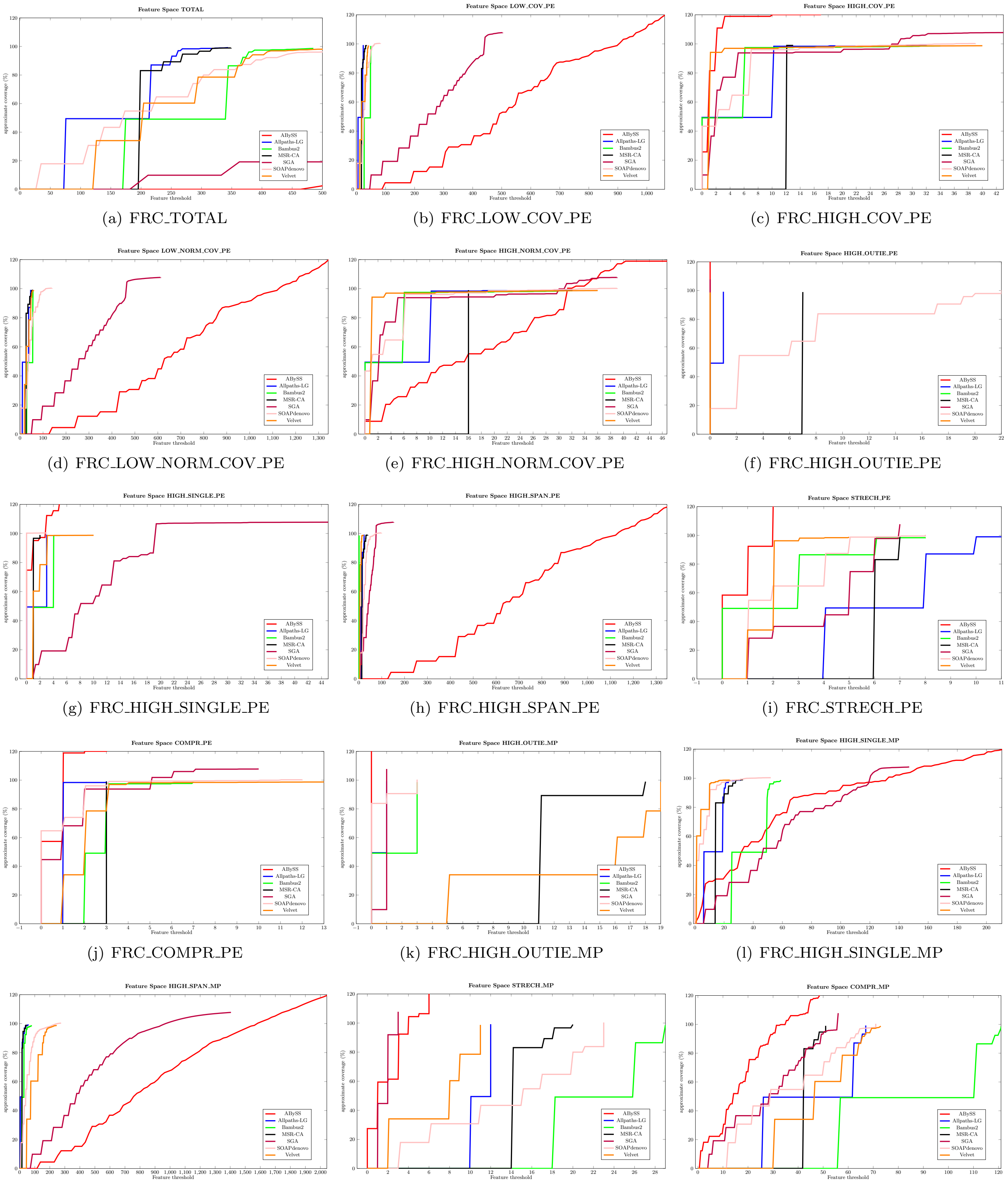

(a) FRC_TOTAL (b) FRC_LOW_COV_PE (c) FRC_HIGH_COV_PE

(d) FRC_LOW_NORM_COV_PE (e) FRC_HIGH_NORM_COV_PE (f) FRC_HIGH_OUTIE_PE

(g) FRC_HIGH_SINGLE_PE (h) FRC_HIGH_SPAN_PE (i) FRC_STRECH_PE

(j) FRC_COMPR_PE (k) FRC_HIGH_OUTIE_MP (l) FRC_HIGH_SINGLE_MP

(m) FRC_HIGH_SPAN_MP (n) FRC_STRECH_MP (o) FRC_COMPR_MP

Figure 10: FRCurves for each feature for Staphilococcus scaffolds

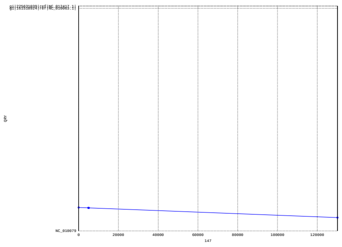

(a) ABySS

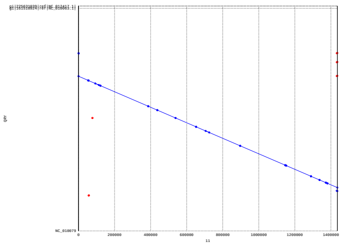

(b) Allpaths-LG

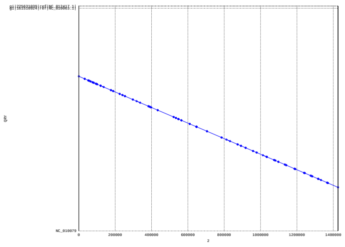

(c) Bambus2

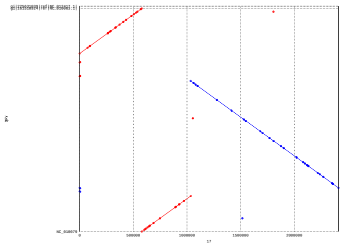

(d) MSR-CA

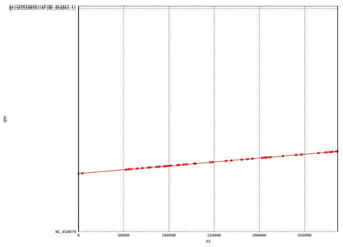

(e) SGA

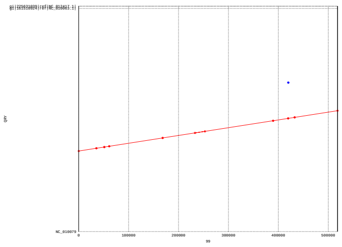

(f) SOAPdenovo

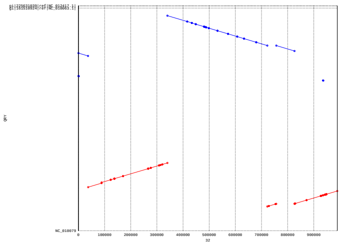

(g) Velvet

Figure 11: dotPlots of longest Scaffold vs Staphylococcus genome

Rhodobacter

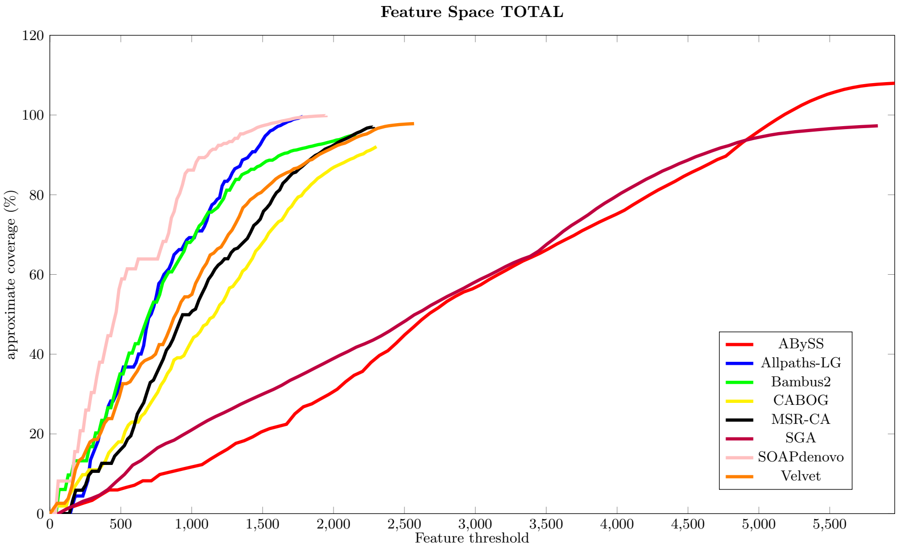

(a) FRCurves for Rhodobacter contigs.

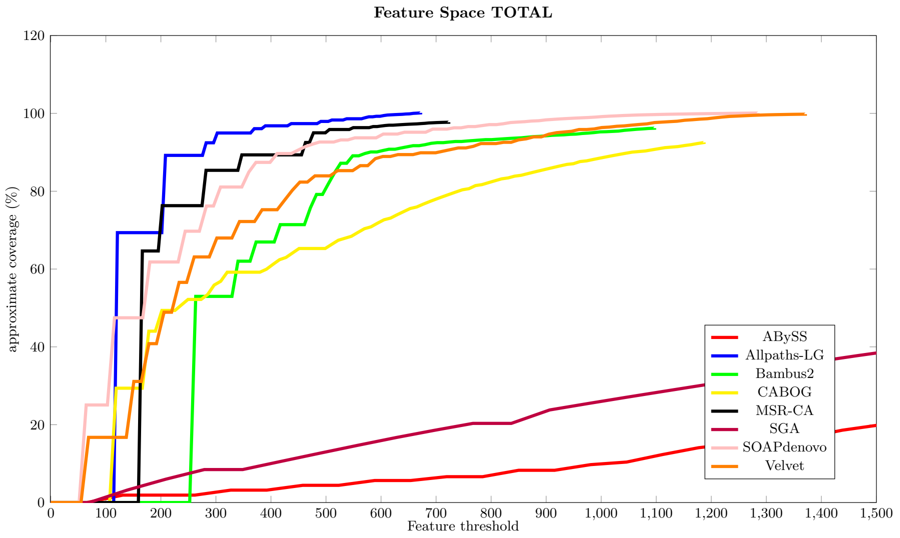

(b) FRCurves for Rhodobacter scaffolds.

Figure 12: FRCurve on Rhodobacter contigs and scaffolds assemblies available on GAGE website.

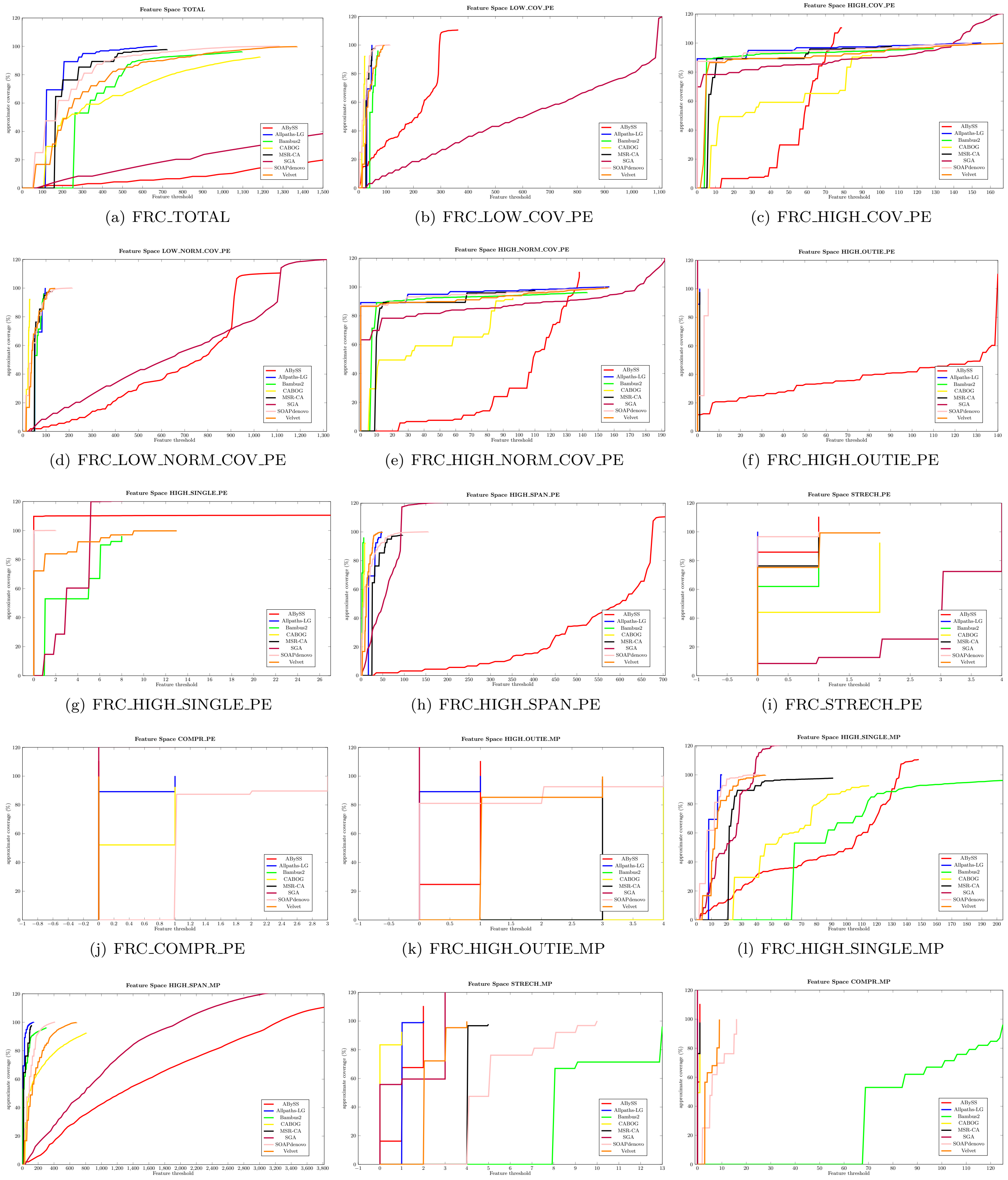

(a) FRC_TOTAL (b) FRC_LOW_COV_PE (c) FRC_HIGH_COV_PE

(d) FRC_LOW_NORM_COV_PE (e) FRC_HIGH_NORM_COV_PE (f) FRC_HIGH_OUTIE_PE

(g) FRC_HIGH_SINGLE_PE (h) FRC_HIGH_SPAN_PE (i) FRC_STRECH_PE

(j) FRC_COMPR_PE (k) FRC_HIGH_OUTIE_MP (l) FRC_HIGH_SINGLE_MP

(m) FRC_HIGH_SPAN_MP (n) FRC_STRECH_MP (o) FRC_COMPR_MP

Figure 13: FRCurves for each feature for Rhodobacter scaffolds

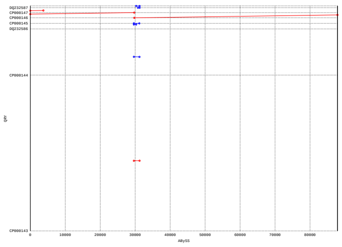

(a) ABySS

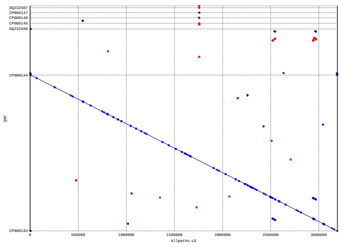

(b) Allpaths-LG

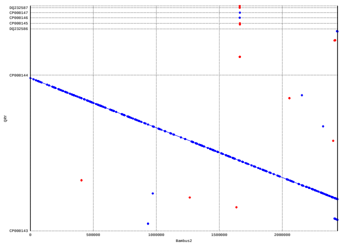

(c) Bambus2

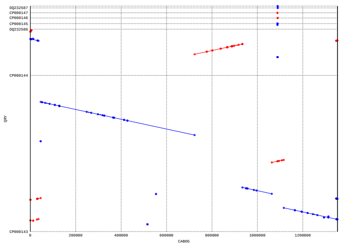

(d) CABOG

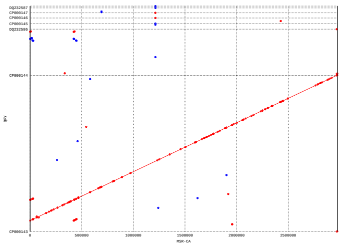

(e) MSR-CA

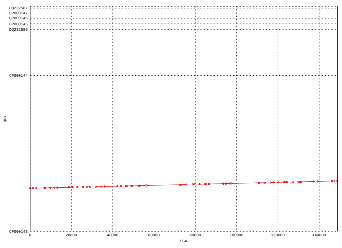

(f) SGA

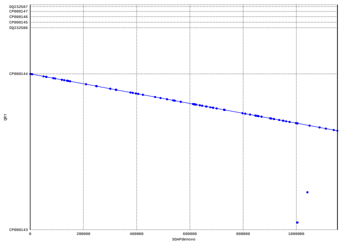

(g) SOAPdenovo

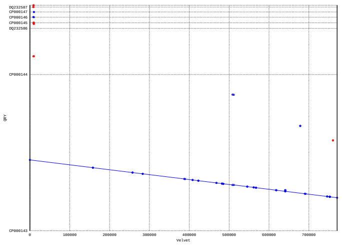

(h) Velvet

Figure 14: dotPlots of longest Scaffold vs Rhodobacter genome

Human Chromosome 14

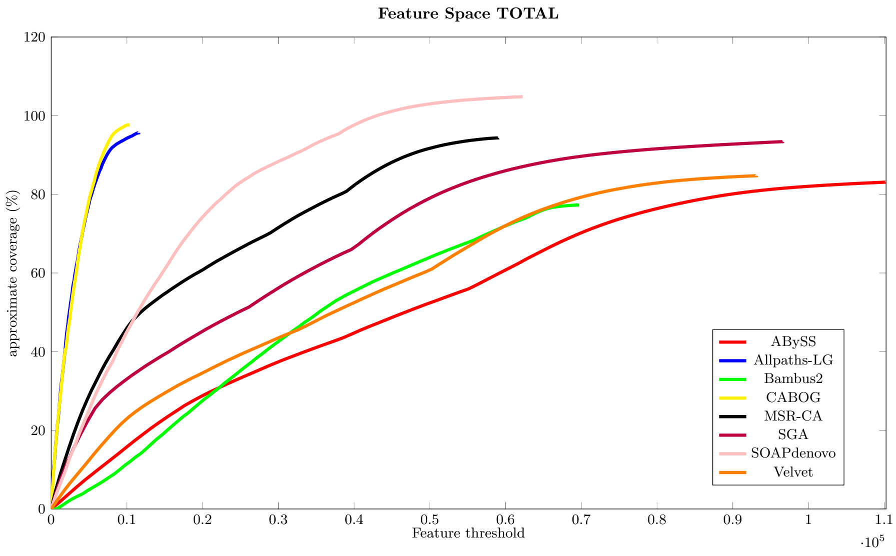

(a) FRCurves for Hc14 contigs.

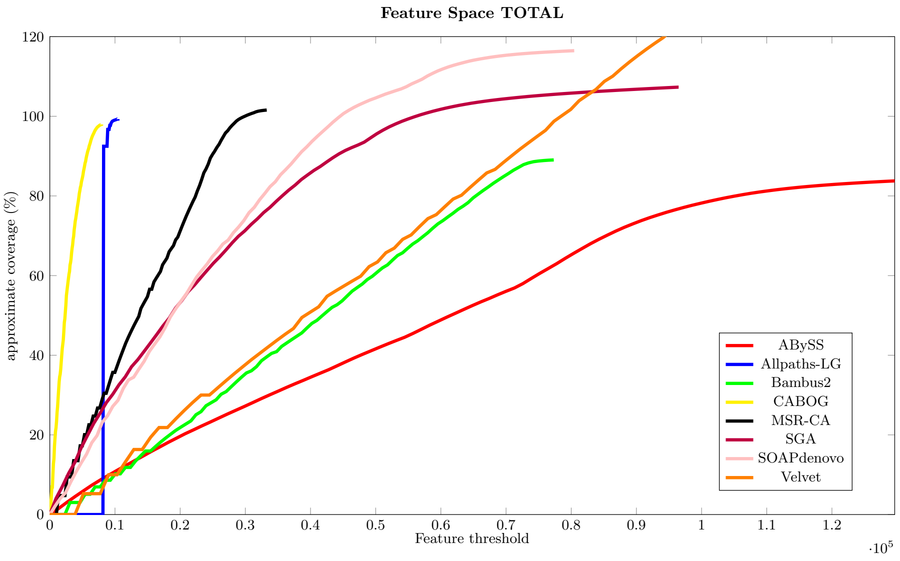

(b) FRCurves for Hc14 scaffolds.

Figure 15: FRCurve on Hc4 contigs and scaffolds assemblies available on GAGE website.

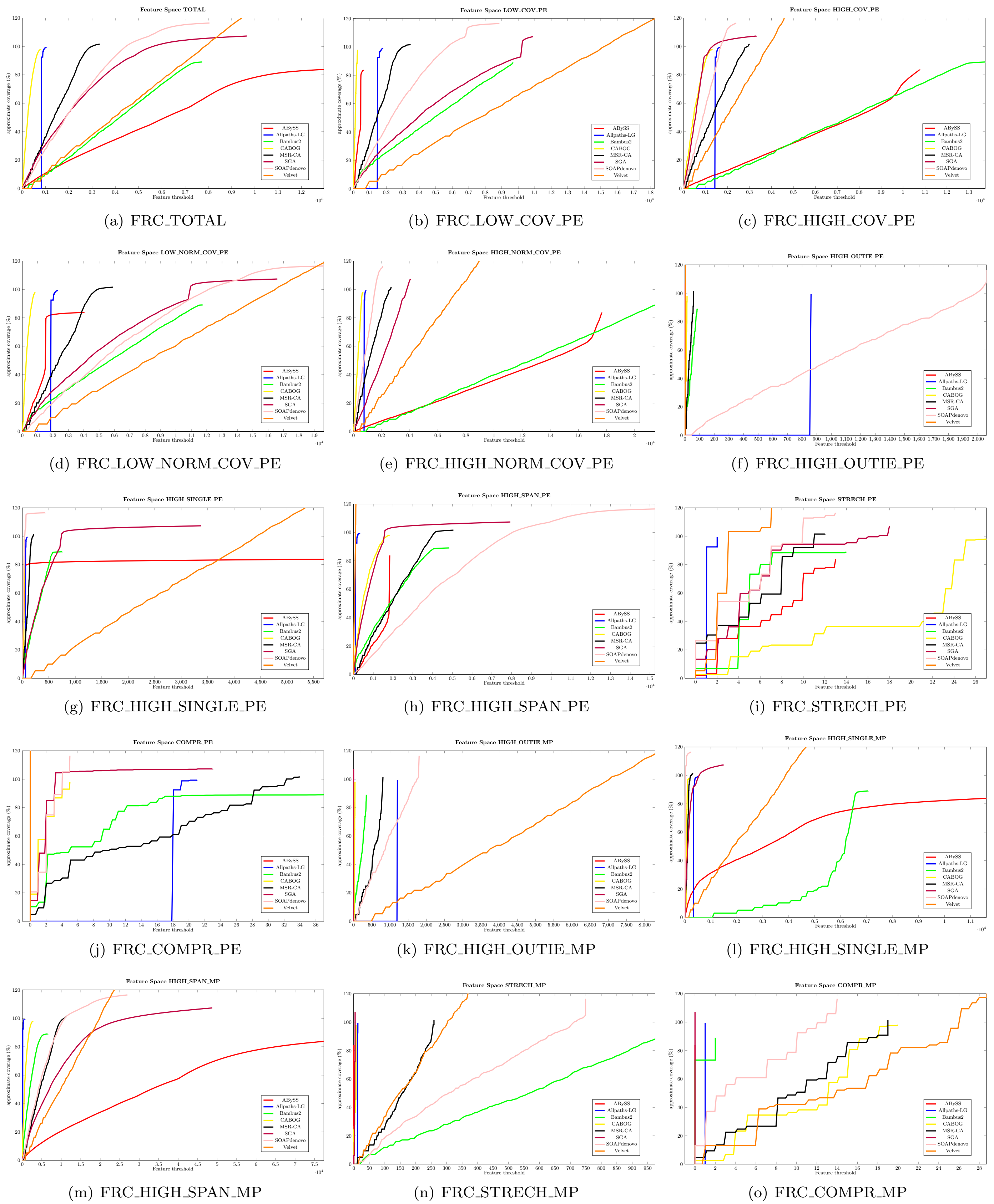

Figure 16: FRCurves for each feature for Hg14 scaffolds

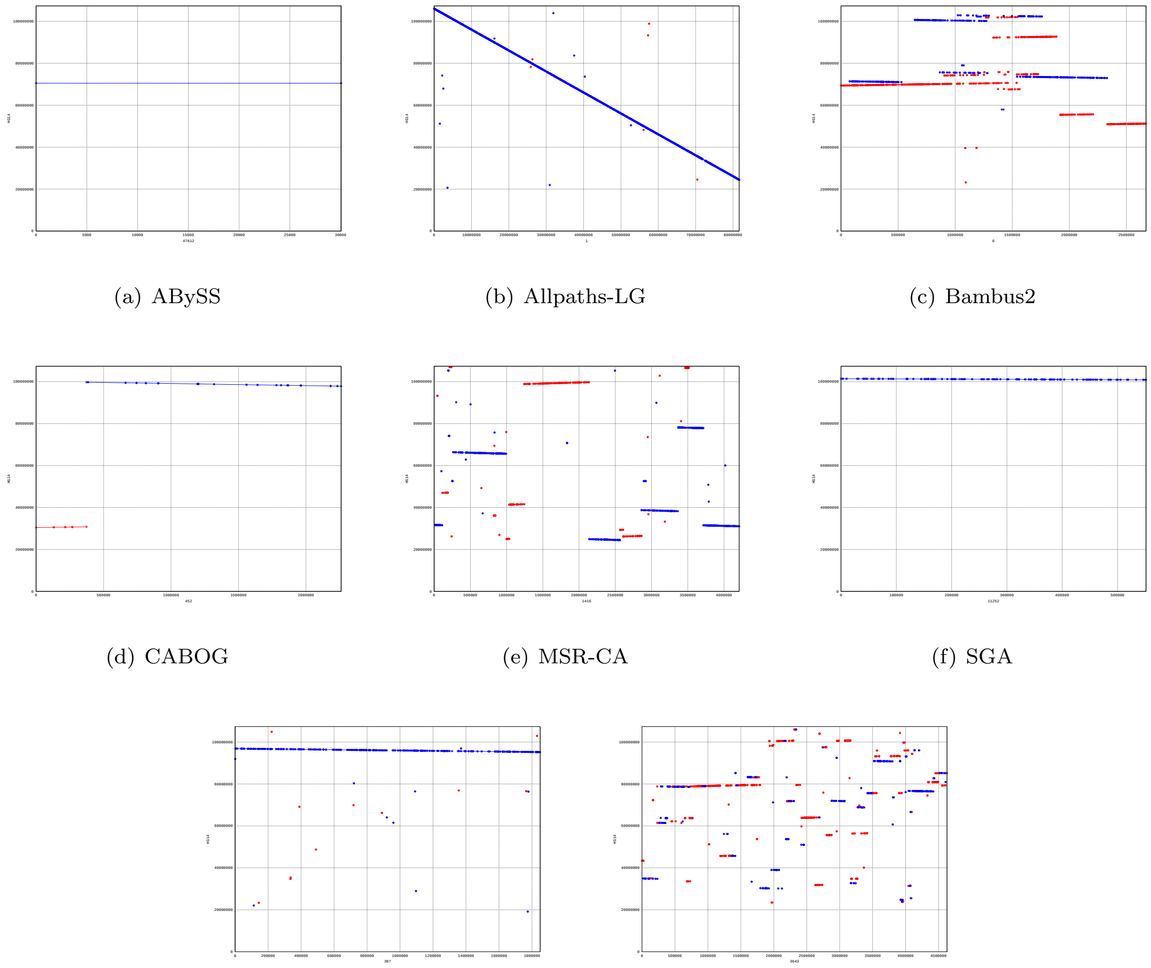

(a) ABySS (b) Allpaths-LG (c) Bambus2

(d) CABOG (e) MSR-CA (f) SGA

(g) SOAPdenovo (h) Velvet

Figure 17: dotPlots of longest Scaffold vs Hg14 sequence

Assemblathon1

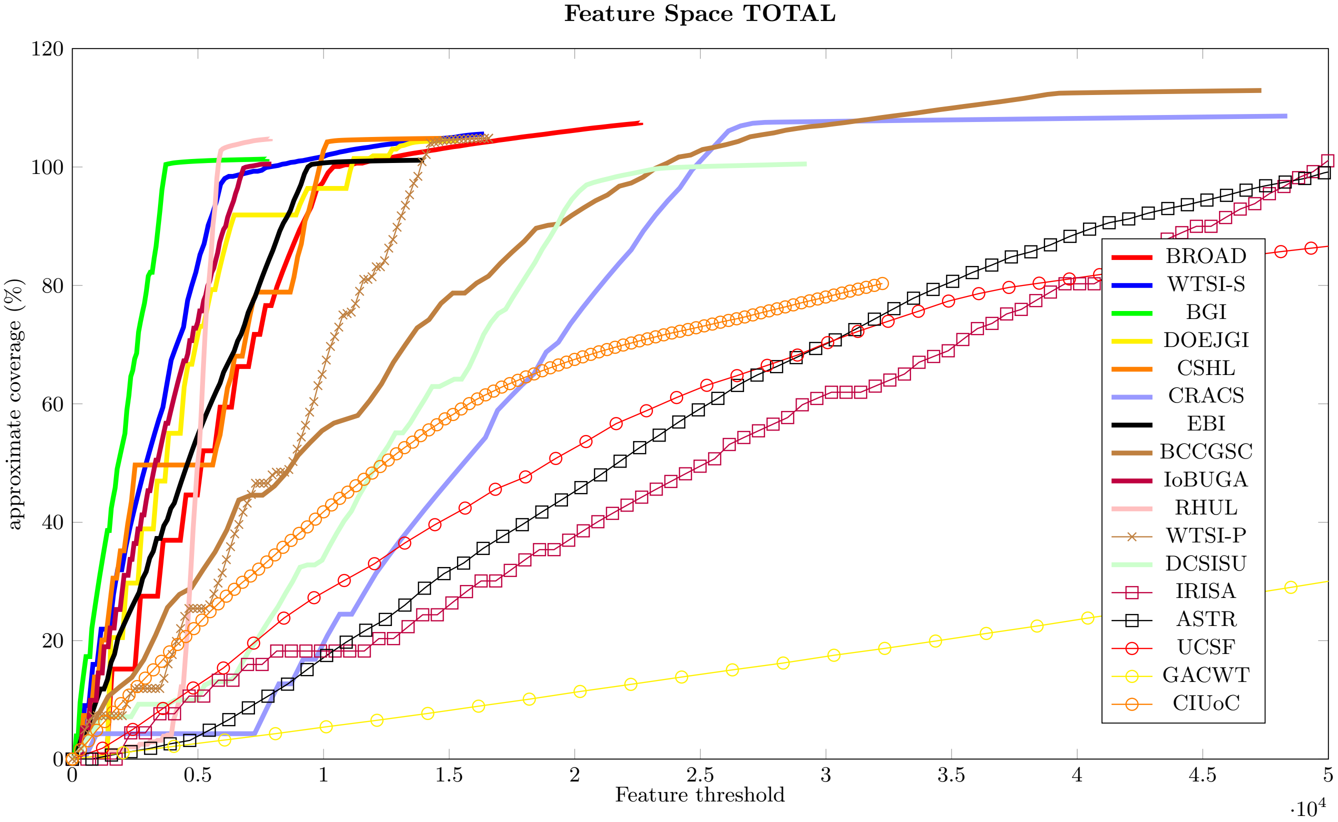

Figure 18: Assmeblathon1 FRCurve

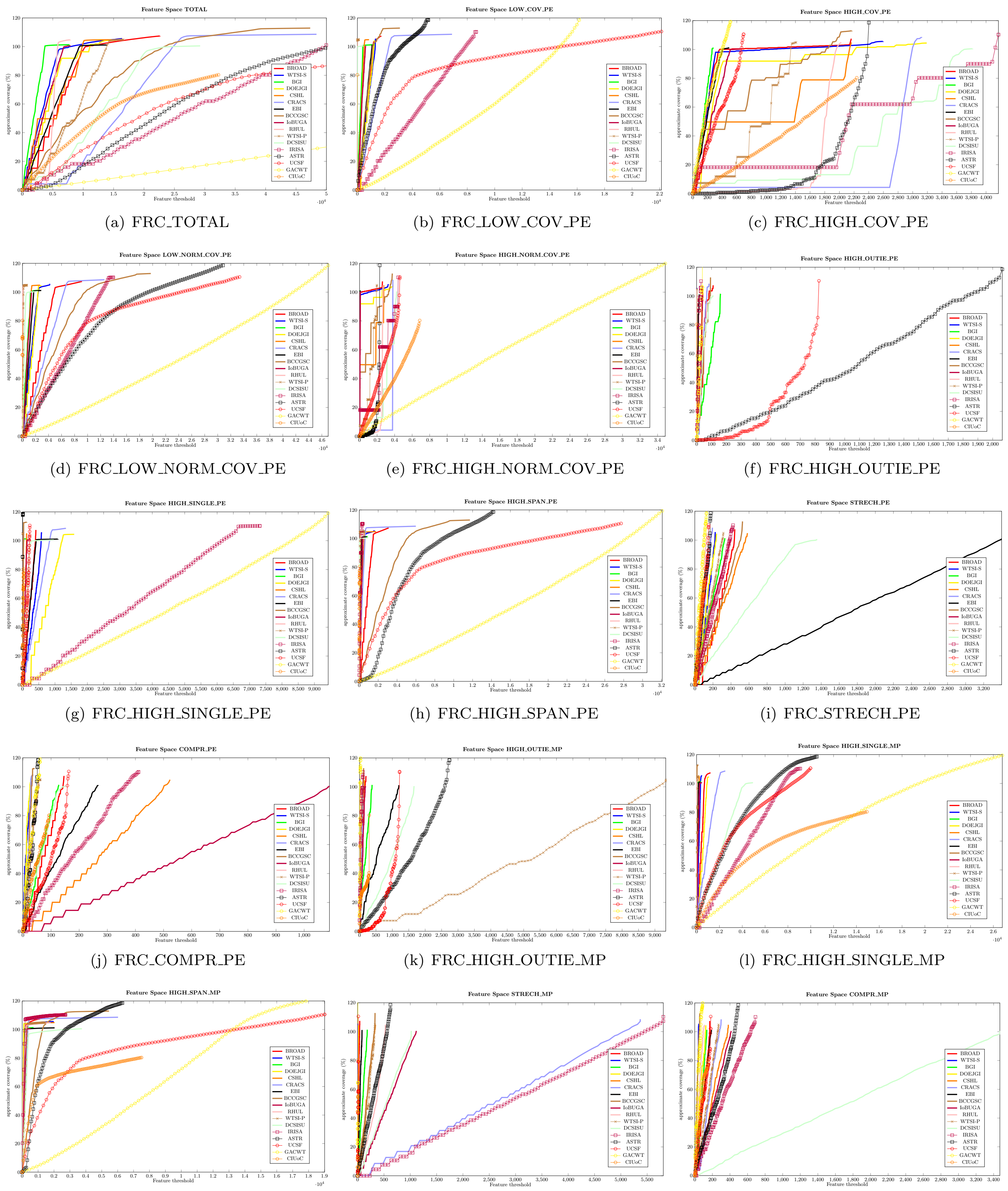

(a) FRC_TOTAL (b) FRC_LOW_COV_PE (c) FRC_HIGH_COV_PE

(d) FRC_LOW_NORM_COV_PE (e) FRC_HIGH_NORM_COV_PE (f) FRC_HIGH_OUTIE_PE

(g) FRC_HIGH_SINGLE_PE (h) FRC_HIGH_SPAN_PE (i) FRC_STRECH_PE

(j) FRC_COMPR_PE (k) FRC_HIGH_OUTIE_MP (l) FRC_HIGH_SINGLE_MP

(m) FRC_HIGH_SPAN_MP (n) FRC_STRECH_MP (o) FRC_COMPR_MP

Figure 19: FRCurves for each feature for Assemblathon1

Assemblathon2

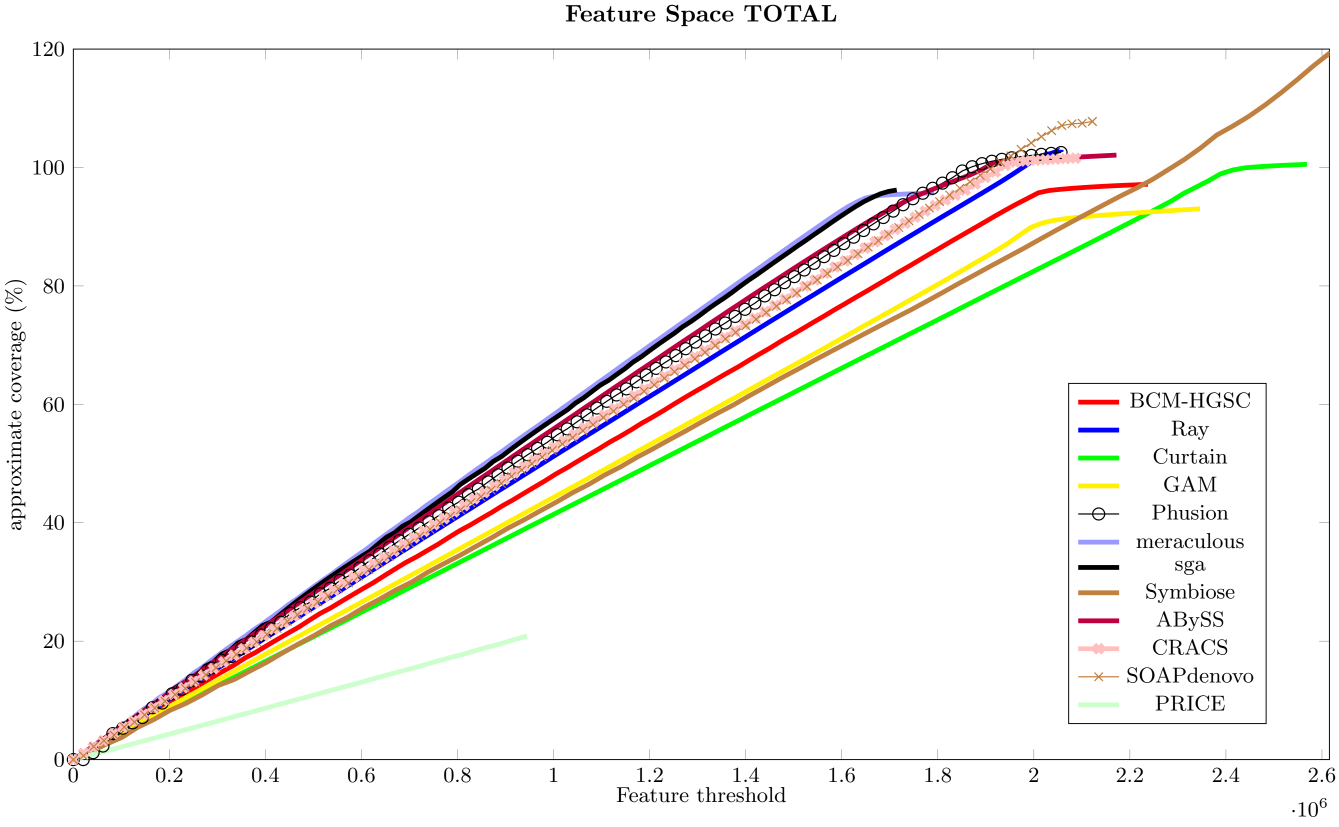

Figure 20: Assmeblathon 2, Snake dataset FRCurves

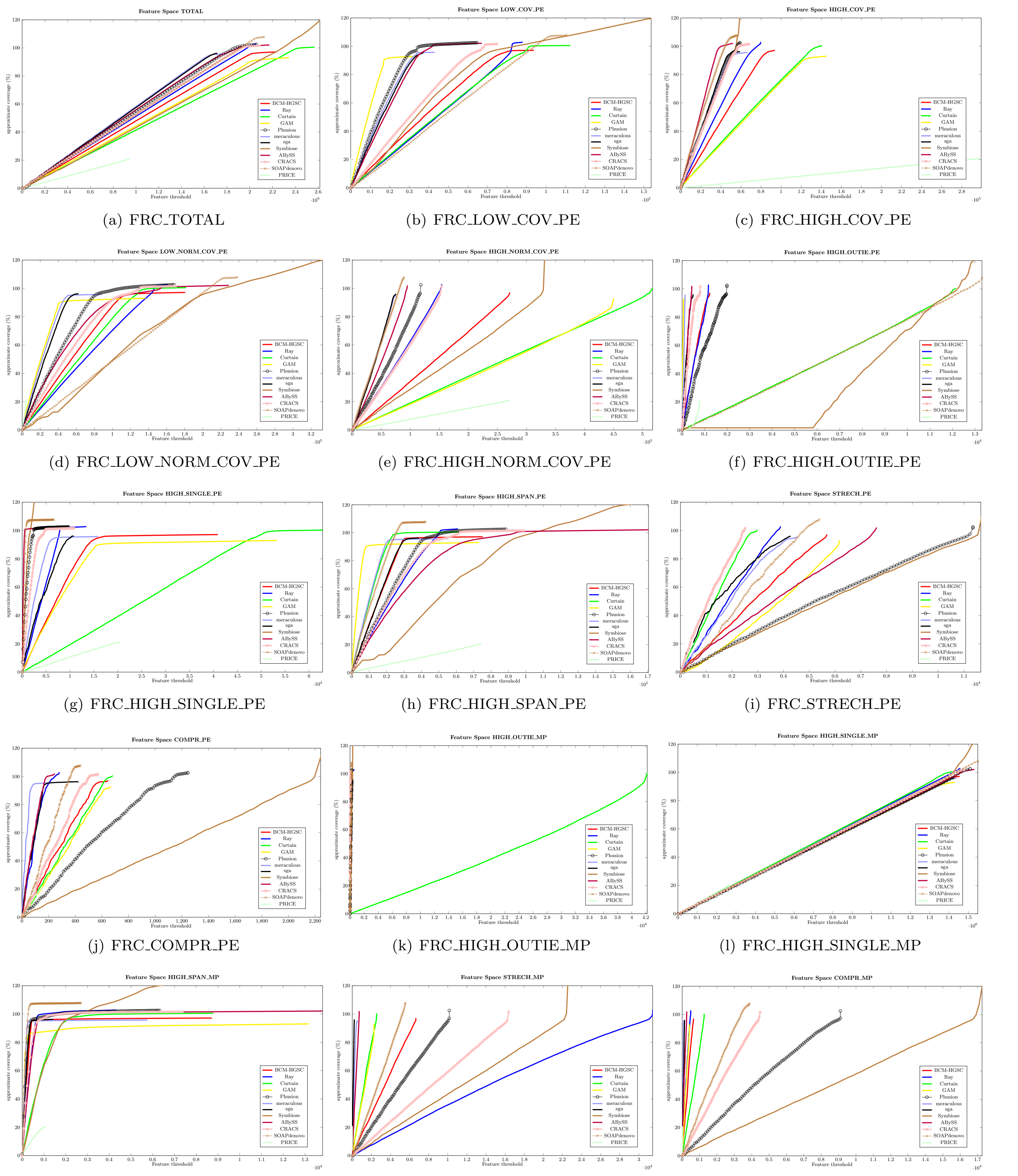

(a) FRC_TOTAL (b) FRC_LOW_COV_PE (c) FRC_HIGH_COV_PE

(d) FRC_LOW_NORM_COV_PE (e) FRC_HIGH_NORM_COV_PE (f) FRC_HIGH_OUTIE_PE

(g) FRC_HIGH_SINGLE_PE (h) FRC_HIGH_SPAN_PE (i) FRC_STRECH_PE

(j) FRC_COMPR_PE (k) FRC_HIGH_OUTIE_MP (l) FRC_HIGH_SINGLE_MP

(m) FRC_HIGH_SPAN_MP (n) FRC_STRECH_MP (o) FRC_COMPR_MP

Figure 21: FRCurves for each feature for Assemblathon2

PCA

We performed a total of 150 assemblies with randomly extracted coverages on three different datasets. We assembled randomly sampled reads from Rhodobacter and Staphilococcus (GAGE dataset) and from Yersinia pestis (downloaded from NCBI: SRX048908) using 8 different assemblers, all run with their default settings: ABySS, Allpaths-LG, CABOG, CLC, MSR-CA, Ray, SGA, and Velvet. Figure 22(a) shows the first principal component plotted against the second. Figure 22(b) is extracted from [9] and shows the same analysis performed on a smaller set of assemblers with features computed with FRC^{amos}.

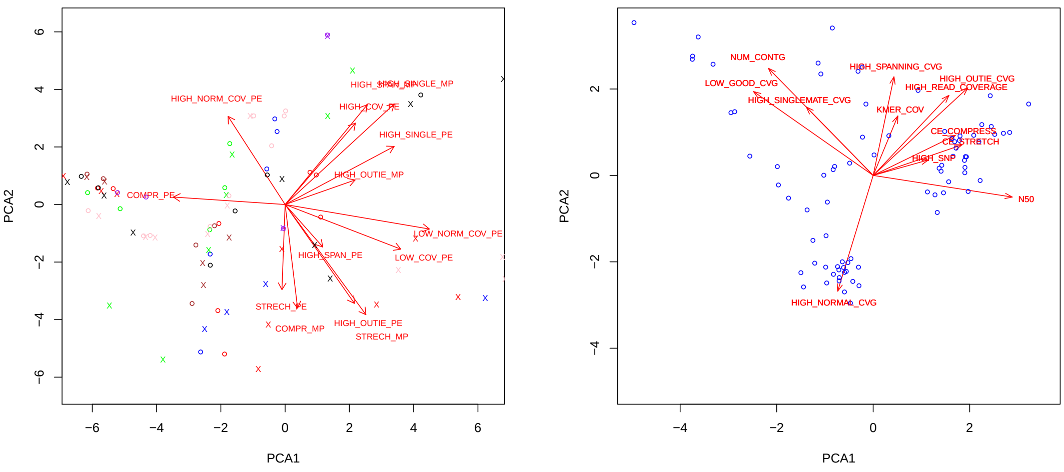

(a) Analysis on FRC^{bam}

(b) Analysis on FRC^{amos}

Figure 22: Figure 22(a) shows PC1 vs PC2 computed on FRC^{bam} features. Contigs are represented by circles, scaffolds are represented by crosses. Each assembler is represented by a different color: (ABySS,red), (Allpaths-LG,blue), (CABOG,green), (CLC,brown), (MSR-CA,pink), (Ray,black), (SGA,purple), and (Velvet,gray). Figure 22(b) shows the same result obtained on a smaller set of assemblies and features with FRC^{amos} (see [9])

As expected, Figure 22(a) shows how assemblers do not form clusters (*i.e.*, they are evenly separated by the features). Such behaviour is in accordance with results reported in [7]: the same assembler can have different results (*i.e.*, different features) even on similar datasets.

Figure 22(b) has been extracted from [9]. It shows PC1 vs PC2 with features computed by FRC^{amos} on similar datasets but by a smaller number of assemblers. Notice that FRC^{amos} does not produce all features computed by FRC^{bam} and vice versa. However, some features are similar (*e.g.*, `HIGH_NORMAL_COV`) and show the same behaviour.

References

[1] Sébastien Boisvert, François Laviolette, and Jacques Corbeil. Ray: Simultaneous Assembly of Reads from a Mix of High-Throughput Sequencing Technologies. *Journal of Computational Biology*, 17(11):101020044546029, October 2010.

[2] Hayan Lee and Michael C. Schatz. Genomic dark matter: the reliability of short read mapping illustrated by the genome mappability score. *Bioinformatics*, 28(16):2097–2105, August 2012.

[3] Heng Li and Richard Durbin. Fast and accurate short read alignment with Burrows-Wheeler transform. *Bioinformatics (Oxford, England)*, 25(14):1754–60, July 2009.

[4] JR Miller, AL Delcher, Sergey Koren, Eli Venter, and B.P. Aggressive assembly of pyrosequencing reads with mates. *Bioinformatics*, 24(24):2818–2824, 2008.

[5] Giuseppe Narzisi and Bud Mishra. Scoring-and-Unfolding Trimmed Tree Assembler: Concepts, Constructs and Comparisons. *Bioinformatics (Oxford, England)*, 27(2):153–160, November 2010.

[6] Adam M Phillippy, Michael C Schatz, and Mihai Pop. Genome assembly forensics: finding the elusive mis-assembly. *Genome biology*, 9(3):R55, January 2008.

[7] S. L. Salzberg, a. M. Phillippy, a. V. Zimin, D. Puiu, T. Magoc, S. Koren, T. Treangen, M. C. Schatz, a. L. Delcher, M. Roberts, G. Marcais, M. Pop, and J. a. Yorke. GAGE: A critical evaluation of genome assemblies and assembly algorithms. *Genome Research*, December 2011.

[8] Francesco Vezzi, Cristian Del Fabbro, Alexandru I Tomescu, and Alberto Policriti. rNA : a Fast and Accurate Short Reads Numerical Aligner. *Bioinformatics (Oxford, England)*, 2011.

[9] Francesco Vezzi, Giuseppe Narzisi, and Bud Mishra. Feature-by-Feature Evaluating De Novo Sequence Assembly. *PLoS ONE*, 7(2):e31002, February 2012.

[10] DR Zerbino and E Birney. Velvet: algorithms for de novo short read assembly using de Bruijn graphs. *Genome research*, 18:821–829, 2008.

[11] Aleksey V Zimin, Douglas R Smith, Granger Sutton, and James a Yorke. Assembly reconciliation. *Bioinformatics (Oxford, England)*, 24(1):42–5, January 2008.
